# Mitigation of ALS Pathology by Neuron-Specific Inhibition of Nuclear Factor Kappa B Signaling

**DOI:** 10.1523/JNEUROSCI.0536-20.2020

**Published:** 2020-06-24

**Authors:** Kallol Dutta, Sai Sampath Thammisetty, Hejer Boutej, Christine Bareil, Jean-Pierre Julien

**Affiliations:** ^1^CERVO Brain Research Centre, Québec City, Québec G1J 2G3, Canada; ^2^Department of Psychiatry and Neuroscience, Université Laval, Québec City, Québec G1V 0A6, Canada

**Keywords:** amyotrophic lateral sclerosis, frontotemporal dementia, IκB suppressor, NF-κB, superoxide dismutase, TDP-43

## Abstract

To investigate the role of neuronal NF-κB activity in pathogenesis of amyotrophic lateral sclerosis (ALS), we generated transgenic mice with neuron-specific expression of a super-repressor form of the NF-κB inhibitor (IκBα-SR), which were then crossed with mice of both sexes, expressing ALS-linked gene mutants for TAR DNA-binding protein (TDP-43) and superoxide dismutase 1 (SOD1). Remarkably, neuronal expression of IκBα-SR transgene in mice expressing TDP-43^A315T^ or TDP-43^G348C^ mice led to a decrease in cytoplasmic to nuclear ratio of human TDP-43. The mitigation of TDP-43 neuropathology by IκBα-SR, which is likely due to an induction of autophagy, was associated with amelioration of cognitive and motor deficits as well as reduction of motor neuron loss and gliosis. Neuronal suppression of NF-κB activity in SOD1^G93A^ mice also resulted in neuroprotection with reduction of misfolded SOD1 levels and significant extension of life span. The results suggest that neuronal NF-κB signaling constitutes a novel therapeutic target for ALS disease and related disorders with TDP-43 proteinopathy.

**SIGNIFICANCE STATEMENT** This study reports that neuron-specific expression of IκB super-repressor mitigated behavioral and pathologic changes in transgenic mouse models of amyotrophic lateral sclerosis expressing mutant forms of either Tar DNA-binding protein 43 or superoxide dismutase. The results suggest that neuronal NF-κB signaling constitutes a novel therapeutic target for amyotrophic lateral sclerosis and related disorders with Tar DNA-binding protein 43 proteinopathy.

## Introduction

Amyotrophic lateral sclerosis (ALS) is characterized by progressive loss of upper and lower motor neurons. ALS evolves with paralysis, and the disease is generally fatal within 2-5 years after the onset of symptoms. Familial ALS, accounting for ∼10% of the ALS cases, is caused by mutations in various genes. Expanded hexanucleotide repeats in C9orf72 account for almost 40% of the familial cases, whereas other mutated genes include superoxide dismutase 1 (*SOD1*), TAR DNA-binding protein (*TARDBP*) encoding TDP-43, fused in sarcoma (*FUS*), and ubiquilin-2 (*UBQLN2*) (Picher-Martel et al., 2015). Abnormal cytoplasmic accumulations of TDP-43 are a common occurrence in degenerating neurons of the CNS in the majority of ALS cases and of tau-negative frontotemporal dementia (FTD) as well as in subsets of Alzheimer's disease and Parkinson's disease (Scotter et al., 2015).

Unexpectedly, TDP-43 was found to directly interact with the p65 subunit of nuclear factor κ B (NF-κB) in CNS samples from ALS individuals (Swarup et al., 2011b) and also from cases with mild cognitive impairment with episodic memory deficits (Ohta et al., 2014). Furthermore, we reported that TDP-43 can serve as coactivator of NF-κB in cultured cells (Swarup et al., 2011b). An involvement of NF-κB in ALS is further supported by the evidence that pharmacological inhibition of this pathway conferred beneficial effects in mouse models of ALS, including attenuation of TDP-43 pathology in motor neurons (Swarup et al., 2011b; Dutta et al., 2017). However, since drug inhibitor NF-κB activity can target systemically different cell types, it has remained unknown whether deregulated NF-κB signaling within neuronal cells contributes to disease pathology and especially formation of abnormal protein aggregates.

NF-κB is a ubiquitously expressed, protean eukaryotic transcription factor that is involved in a multitude of physiological functions. NF-κB acts as transcription activator for several genes associated with inflammation, and it is considered one of the key modulators for inflammatory pathways. NF-κB remains sequestered in cellular cytosol as a complex composed of either of two classes of proteins: Class 1 containing NF-κB1 (p50/p105) and NF-κB2 (p52/p100), and Class 2 containing RelA (p65), RelB, and c-Rel. At basal state, NF-κB proteins are trapped in cell cytoplasm by association with inhibitory (I) κB proteins, including IκBα, IκBβ, IκBε, and Bcl-3 (Ghosh and Karin, 2002), their activation being dependent on a series of sequentially activated kinases. When classically activated, a kinase complex (IκB-kinase or IKK complex) phosphorylates IκBα on Ser32 and Ser36, leading to its ubiquitination and proteasomal degradation (Viatour et al., 2005). As a result, NF-κB is released and translocates into the nucleus to mediate its functions. NF-κB can undergo post-translational modifications, such as phosphorylation or acetylation, before nuclear translocation.

To investigate the role of neuronal NF-κB signaling in ALS pathogenesis, we generated transgenic mice with neuron-specific expression of a super-repressor form of the NF-κB inhibitor IκB (IκBα^S32A, S36A^). The IκBα super-repressor mice were further cross-bred with three different transgenic mouse models of ALS: two transgenic lines expressing human TDP-43 with point mutations A315T and G348C and a mouse model expressing SOD1 with G93A mutation. The hTDP-43^A315T^ and hTDP-43^G348C^ transgenic mice exhibit during aging abnormal cytoplasmic accumulations of human TDP-43, substantial motor neuron loss as well as motor and cognitive deficits (Swarup et al., 2011a). On the other hand, the well-established SOD1^G93A^ mice replicate ALS disease phenotypes with accumulations of misfolded SOD1 species, severe motor neuron loss resulting in short life span (Dutta et al., 2018). Here, we report that neuron-specific expression of IκB-SR ameliorated behavioral and pathologic phenotypes in three mouse models of ALS carrying either human mutated TDP-43 or SOD1 transgenes. The results suggest that neuronal NF-κB signaling constitutes a novel therapeutic target for ALS disease.

## Materials and Methods

### 

#### 

##### DNA constructs, generation of transgenic mice, and genotyping

The DNA of neurofilament H (NFH) promoter was amplified by PCR using the genomic DNA purified from mouse tail. The following primers were used for the NFH amplification: 5′ primer: 5′-GGGACGACGGTACCCTAGGACATTCTGGGCTGAGATC-3′ and 3′ primer: 5′-GGGACGACGTCGACCAGCGGAGCGGGAGTGCGGGGCT-3′. A 2.8 kb fragment was purified on agarose gel and subcloned into KpnI-SalI restriction sites of pBluescript KS^+^ (pBSKS-NFH promoter). Flag-EGFP fragment was obtained by PCR using pEGFP-N3 plasmid as template (Clontech). The following primers were used for the Flag-EGFP fragment amplification: 5′ primer: 5′-GGGACGACCTGCAGGAGGCAGCATGGACTACAAAGACGACGACGACAAGGTGAGCAAGGGCGAGGA-3′ and 3′ primer: 3'-GGGACGA CGGATCCCTTGTACAGCTCGTCCATGC-3′. The obtained fragment was introduced into corresponding restriction sites of pBSKS-NFH promoter plasmid. The human IκBα (SS32, 36AA) cDNA was amplified by PCR using the plasmid PCMV4-3HA/IκBα (SS32, 36AA) (Addgene, plasmid #24143) (Sun et al., 1996) as template. The following primers were used for the PCR amplification: 5′ primer: 5′-GGGACGACGGATCCTTCCAGGCGGCCGAGCGCCCCCAGG-3′ and 3′ primer: 5′-GGGACGACGCGGCCGCTCATAACGTCAGACGCTGGCCTCCA-3′.

A 953 bp BamHI/NotI fragment corresponding to the human IκBα (SS32, 36AA) cDNA was introduced into pBSKS recombinant vector. The integrity of the final construct was verified by sequencing.

For the generation of the transgenic mice, a BssHII-BssHII DNA fragment of 4.9 kb was isolated on agarose gel for microinjection. The transgenic mice were viable and did not develop overt phenotypes and were genotyped by PCR amplification. For genotyping, a 329 bp fragment from the EGFP gene is amplified from the NFH-IκBα (SS32, 36AA) transgenic mice (henceforth referred to as IκB super-repressor or IκB-SR mice) and not from the WT mice. The PCR was performed on ear punch samples using the 5′ EGFP-GEN primer: 5′-AGTTCATCTGCACCACCGGC-3′ and the 3′ EGFP-GEN primer: 5′-CGGCCATGATATAGACGTTG-3′.

These IκB-SR mice were further cross-bred with transgenic mice bearing genomic fragment expressing familial ALS-linked mutant TDP-43 (G348C and A315T), which have been previously generated, characterized, and reported from our laboratory (Swarup et al., 2011a). The double-transgenic mice, along with their littermate single-transgenic and nontransgenic mice, have been used for this current study. The sex ratio was kept close to 1:1 for all genotype groups. All experimental procedures were approved by the Laval University Animal Care Ethics Committee (protocol #2016–060-1 and 2016-060-2) and were in accordance with The guide to the care and use of experimental animals of the Canadian Council on Animal Care.

##### Validation of IκB super-repressor in vitro

To confirm the efficacy of the super-repressor in inhibiting NF-κB activation, the sequence was cloned into pcDNA3 vector and transfected with lipofectamine into motor neuron-like NSC-34 cells, seeded in 35 mm plates in duplicate (2 µg of plasmid DNA per plate). Nontransfected cells from the same passage served as control. At 48 h after transfection, cells were treated either with recombinant mouse TNF-α (40 ng/ml) or vehicle and incubated for a further 6 h. After incubations, media was discarded, the cells were harvested, lysed with RIPA buffer (10 mm Tris-Cl, pH 8.0, 1 mm EDTA, 0.5 mm EGTA, 1% Triton X-100, 0.1% sodium deoxycholate, 0.1% SDS, 140 mm NaCl, protease and phosphatase inhibitors), and probed for phospho-NF-κB and total NF-κB.

##### Motor performance test

To test motor coordination, mice were trained to run on an accelerating rotarod for 1 week (three trials on alternate days) before the beginning of recording. The experimental paradigm for SOD1^G93A^, IκB-SR;SOD1^G93A^, and their littermate controls was set at an initial speed of 0 rpm with acceleration of 0.1 rpm/s, whereas for the TDP-43 mutant mice, along with the double-transgenics with IκB-SR and their littermate controls, it was 3 rpm with acceleration of 0.2/s. The cutoff was set at 300 s for all mice. Mice were subjected to three trials per session every week, and the longest latency to fall from the rotating rod was recorded. Hindlimb strength of SOD1^G93A^, IκB-SR;SOD1^G93A^, and their littermate controls was further assessed by the grid-hang test. Briefly, the mice were placed on a wire grill, which was then turned over gently, allowing the animal to hang from the grill. The time elapsed before the hindlimbs of the mice lets go of the grill was recorded with a cutoff time set at 90 s.

##### Behavioral analyses

TDP-43 pathology is also associated with frontotemporal dementia, which is replicated in our transgenic animal models. To assess the effect of suppression of NF-κB activation on memory functions, transgenic mice from the IκB-SR and TDP-43 mutant backgrounds (i.e., IκB-SR;TDP-43^A315T^, IκB-SR;TDP-43^G348C^, TDP-43^A315T^, TDP-43^G348C^, and IκB-SR) and their nontransgenic littermates were subjected to novel object recognition tests at 6 and 12 months of age. Briefly, on day 1 of the test, the mice were allowed to habituate in the experimental cage for a fixed amount of time (5 min). On day 2, two similar objects (same shape and color) were placed at two predetermined spots in the cage, and the mice were allowed to explore either of them, *ad libitum*, for the same duration of time. On day 3, one of the objects was replaced with a novel object (different shape and color), and the exploratory time of the mice at both the familiar and the novel object was recorded by an observer, who was blinded to the genotype of the animals. The results were expressed as percentage of time spent in exploring the novel object relative to the total time spent at both the objects. This test was first performed when the mice were 6 months of age and repeated when they were 12 months old with an entirely different set of objects.

The passive avoidance task is an aversive (emotional) conditioning paradigm whereby the innate preference for the dark compartment of a test apparatus by rodents are suppressed following exposure to an inescapable noxious stimuli (Ögren and Stiedl, 2010). It is a common learning and memory function test that is commonly used to test for cognitive decline in models of neurodegeneration. On day 1 of the test, the mice were allowed to familiarize with the surroundings. Initially, the mice were placed in a lighted chamber that was separated from a dark chamber by a time-controlled door. After a specific time (30s), the door was programmed to open and based on the natural light avoidance tendency of the mice, the time taken to enter the dark chamber was noted. On day 2, the mice were allowed to remain in the lighted chamber for the same duration of time, but the dark chamber was rigged to deliver an electric shock (0.5 mA for 5s) via the footpad. Once the mice entered the dark chamber and received the footpad shock, they were confined in there for a further 60 s so as to reinforce the memory of the noxious stimuli. On day 3, the mice were once again placed in the lighted chamber, but the door connecting it with the dark chamber was opened after 5 s. The time taken by the mice to reenter the dark chamber was noted, again by an observer blinded to the genotypes. A significant delay or refusal to reenter the dark chamber was considered to be indicative of learned response against the noxious stimuli. Because of the stressful nature of the passive avoidance task, the mice were subjected to only one trial at 12 months of age.

##### Analysis of clinical symptoms of SOD1^G93A^ mice

The SOD1^G93A^ model is an acute model of familial ALS. The onset of disease was estimated to correspond with the time when mice started to exhibit a decline of body weight after reaching a peak. End stage was defined as the loss of righting reflex (the age when the animal could not right itself within 30 s when placed on its side or when a loss of >30% peak body weight was recorded). A 3 point scoring system based on hindlimb reflexes, where nonsymptomatic animals (hindlimbs extended forming an angle of 120 degrees) was assigned a score of 3, whereas end stage animals (showing loss of reflex with hindlimbs paws held close to the body and unable to walk) being assigned a score of 0 were used to monitor the progression of disease in SOD1^G93A^ mice (Urushitani et al., 2006). The same approach was also adopted for the IκB-SR;SOD1^G93A^ and littermate controls. This reflex scoring was done by animal facility technicians who were blinded for genotypes but had experience in grading paralysis in mice. The body weights of these animals were also recorded, weekly before advent of symptoms and daily after symptoms. The decline in body weight also serves as a clinical indicator for rate of disease progression in this model.

##### Histology and immunofluorescent staining of tissue sections

Animals were anesthetized by intraperitoneal administration of sodium pentobarbital (10 mg/kg body weight) and transcardially perfused with ice-cold saline followed by fixation with cold solution of 4% PFA. Spinal cords were excised carefully and stored overnight in 4% PFA solution. On the following day, the tissues were transferred to a 30% sucrose solution and stored for 24-48 h before sectioning with a microtome. Spinal cord sections (20 µm thickness) were mounted on glass slides and allowed to adhere in a vacuum chamber. The slides were washed with 1× TBS (50 mm Tris-Cl, pH 7.5; 150 mm NaCl) and permeabilized with 1× TBS-0.1% Triton X-100. The slides were then blocked for 2 h at room temperature with 10% solution of normal goat serum (Invitrogen) in 1× TBS-0.1% Triton X-100. While staining for FLAG using anti-FLAG M2 antibody, blocking was done with a 5% solution of nonfat milk in 1× TBS-0.1% Triton X-100, as other blocking agents (goat serum or 10% albumin) failed to reduce nonspecific antibody binding. After blocking, sectioned were incubated overnight at 4°C with appropriate primary antibodies (for details, see Table 1). On the next day, the slides were washed extensively with 1× TBS followed by incubation with appropriate fluorochrome-conjugated secondary antibodies (AlexaFluor; Thermo Fisher Scientific) at room temperature in a dark chamber, for 2 h. After washes, slides were briefly incubated with DAPI (Thermo Fisher Scientific) followed by another incubation (10 min) with 0.05% Sudan Black B solution in 70% ethanol to quench autofluorescence. After repeated washes, slides were mounted with Fluoromount-G (Electron Microscopy Sciences) and visualized under an Apotome or a Leica DM5000B microscope (Carl Zeiss).

**Table 1.** Details of antibodies used for immunoblotting, immunoprecipitation, and immunofluorescence

Antibody against	Dilution for immunoblotting/immunoprecipitation	Dilution for immunofluorescence	Company
Actin	1:15,000		Sigma Millipore
Atg-5		1:250	Novus Biologicals
Beclin-1	1:1000		Novus Biologicals
ChAT		1:500	Sigma Millipore
FLAG-M2	1:2500 2 µg/300 µg of total proteina^a^	20 µg/ml	Sigma Millipore
GAPDH	1:2500		Santa Cruz Biotechnology
GFAP		1:300	Cell Signaling Technologies
GFP	1:1000	1:300	Thermo Fisher Scientific
Iba-1		1:500	Wako Chemicals
IkBα	1:2000		Santa Cruz Biotechnology
Phospho-IκBα	1:1000		Cell Signaling Technologies
LC3-II		1:250	Novus Biologicals
NeuN		1:500	Cell Signaling Technologies
NF-κB	1:1000 1.5 µg/300 µg of total proteina^a^		Santa Cruz Biotechnology
Phospho-NF-κB (Ser 536)	1:1000		Cell Signaling Technologies
SOD1 mAb (misfolded; clone B8H10)	1 μg/300 μg total proteina^a^		Generated from hybridoma in the laboratory
SOD1 pAb	1:5000		Enzo Life Sciences
Pan TDP-43	1:2500		Proteintech
Human TDP-43	1:1000		Thermo Fisher Scientific
Human TDP-43		1:250	Abnova

*^a^*For immunoprecipitation.

10.1523/JNEUROSCI.0536-20.2020.t1-1 Table 1-1 Comparison between reflex scoring of IκB-SR;SOD1^G93A^ vs SOD1^G93A^ (Figure 8D). Download Table 1-1, DOCX file

##### Image analysis

Image analysis was performed using the Fiji software (freely downloadable from https://imagej.net/Fiji). For measuring cellular fluorescence, signal intensity methodology described by Arqués et al. (2012), was used, with minor modifications. Briefly, the ROIs (cell boundary or nucleus) were marked using the freehand selection tool. The area of the ROI and integrated density was measured along with the mean fluorescence of background. The corrected total fluorescence was calculated using the following formula: Corrected total fluorescence = Integrated density – (Area of ROI × Mean fluorescence of background readings). GFAP and Iba-1 signal intensities from ventral horns of spinal cord was measured by method previously described (Dutta et al., 2017). Microglial branching pattern analysis was performed by methods described by Young and Morrison (2018), with minor modifications. Briefly, *z*-stack images of spinal cord sections at 20× magnification were captured and maximum intensity projection images were generated. The images were then adjusted for optimal brightness and contrast followed by application of an Unsharp mask (pixel radius 3; mask weight 0.6). The images were then despeckled to remove salt-and-pepper noise followed by their conversion to binary images by threshold adjustment. The binary images were subjected to another round of despeckling followed by outlier removal (pixel radius 2; threshold 50). Finally, the binary images were skeletonized and saved as separate files. At random, skeleton images of cells (at least 24 from 3 animals) were reopened with Fiji and run through the AnalyzeSkeleton (2D/3D) plugin and checking the Branch Information box. The generated data were saved in an Excel spreadsheet. Data were sorted by endpoint voxels from largest to smallest and the cutoff length for undesired fragments was set at 0.5 µm. The number of branching per cell and total length of branches per cell were tabulated in a separate file, which were used for phenotypic analysis (Young and Morrison, 2018).

##### Immunoblot analysis

Animals were anesthetized by the method described above. Tissues (brain, spinal cord, kidney, liver, spleen) were excised quickly and snap-frozen in liquid nitrogen followed by storage in −80°C freezer until further use. Hippocampus from mice brain was dissected following protocol demonstrated in the *European Journal of Neuroscience*'s protocol video (retrieved from EJNeuroscience's YouTube channel: https://www.youtube.com/watch?v=Upf15CB29V4). In general, tissues were homogenized using chilled RIPA buffer, sonicated, and centrifuged for 20 min at 12,000 × *g* at 4°C. The supernatant (soluble fraction) was removed, and the pellets were washed 3 times with RIPA buffer followed by resuspension in urea (6 M) buffer, which served as insoluble fraction. Spinal cords from SOD1 mice carrying the SOD1 mutation were homogenized in a lysis buffer (TGNT buffer) containing 50 mm Tris-HCl, pH 7.4, 100 mm NaCl, 10% glycerol, and 1% Triton X, sonicated followed by centrifugation for 20 min at 9000 × *g* at 4°C.

Proteins from tissue lysates were electrophoretically separated on SDS-polyacrylamide gel and transferred onto PVDF membranes. The membranes were then blocked with 7% nonfat milk in 1× PBS containing 0.1% Tween-20 followed by incubation with primary antibodies (for details, see Table 1) diluted in 1% BSA solution. After incubation, blots were washed and reincubated with appropriate secondary antibodies conjugated with HRP. Signal was acquired by exposing chemiluminescent reagent-treated membranes to X-ray films (Biomax MR, Kodak) or in a ChemiDoc MP Imaging System (Bio-Rad). Band intensities were analyzed using Fiji and normalized to housekeeping proteins Actin or GAPDH. To visualize total proteins on gels for normalizing insoluble proteins, Coomassie staining protocol was used. Briefly, after electrophoresis, gels were incubated in Coomassie stain (0.1% Coomassie R-250 in 50% methanol, 10% acetic acid, 40% H_2_O) and incubated at room temperature for 1 h. The gel was then incubated in destainer (5% methanol, 7.5% acetic acid, 87.5% H_2_O) with changes until a clear background was achieved.

##### Immunoprecipitation of FLAG fusion proteins

For proteomic confirmation of IκB-SR transgene expression, spinal cords from IκB-SR transgenic mice were lysed in RIPA buffer supplemented with protease inhibitor cocktail. The homogenates were centrifuged at 20,000 × *g* for 10 min at 4°C, and the supernatants were added onto paramagnetic Protein A/G beads (Dynabeads; Invitrogen) precoated with anti-FLAG M2 antibody (Table 1). The sample-bead mixtures were incubated overnight at 4°C on an orbital shaker. The following day, the beads were washed and samples were eluted with Laemmli sample buffer and subjected to SDS-PAGE and immunoblotting using anti-IκBα antibody.

##### Coimmunoprecipitation for P65 NF-κB and hTDP-43

Protein A/G-coated paramagnetic beads (Dynabeads; Invitrogen) were coated with anti-P65 NF-κB antibody (Table 1) at the recommended concentration (1.5 µg) for 2 h at room temperature. After washings to remove unbound IgG, the beads were incubated with spinal cord lysates in RIPA buffer containing 300 µg proteins, at 4°C, overnight. On the following day, the beads were washed, proteins eluted, and separated on a 10% SDS-polyacrylamide gel followed by electrophoretic transfer onto a PVDF membrane. The membranes were subsequently blocked with 5% BSA and incubated overnight with antihuman TDP-43 antibody. Immunodetection was done using goat antirabbit HRP-labeled secondary antibody (Jackson ImmunoResearch Laboratories) and visualized on X-ray film (Biomax MR1; Kodak) following exposure to enzyme chemiluminecent reagent (Thermo Fisher Scientific).

##### Immunoprecipitation for misfolded SOD1

Antibody against misfolded SOD1 (clone B8H10) was purified from serum-free culture media of hybridomas. The media was treated with a saturated solution of ammonium sulfate, and the salted-out proteins were subsequently dialyzed to obtain the antibody. Immunoprecipitation was performed using protein A/G-coated paramagnetic beads (Dynabeads; Invitrogen) according to a previously published protocol with minor modifications (Gros-Louis et al., 2010). Briefly, the beads were first coated with the monoclonal anti-misfolded SOD1 antibody (clone B8H10; 1.0 µg of antibody per 40 µl of beads) for 2 h at room temperature. Following washes to remove unbound antibodies, the beads were incubated with volumes of spinal cord lysates corresponding to 100 µg of total protein, at 4°C, overnight. On the following day, the beads washed, proteins were eluted and separated on a 14% SDS-polyacrylamide gel and then transferred electrophoretically to PVDF membrane. After blocking with 5% nonfat milk, membranes were incubated with polyclonal primary antibody against SOD1 (Table 1). Immunodetection was performed with a goat antirabbit HRP-labeled secondary antibody (Jackson ImmunoResearch Laboratories) and visualized on X-ray film (Biomax MR1; Kodak) following exposure to enzyme chemiluminescent reagent (Thermo Fisher Scientific).

##### Statistical analysis

All statistical analysis was done using GraphPad Prism (version 7.01). Before analyzing data from multiple groups, the normal distribution of the data was checked using the D'Agostino & Pearson omnibus normality test. Comparisons between multiple groups with normally distributed data were done using one-way ANOVA with Tukey's post-test. In cases of non-normal distribution, data were analyzed using Kruskal-Wallis test along with Dunn's post-test. Comparisons between two groups were done by Student's *t* test with Welch's correction.

## Results

### Neuron-specific expression of IkB super-repressor

**Figure 1.**
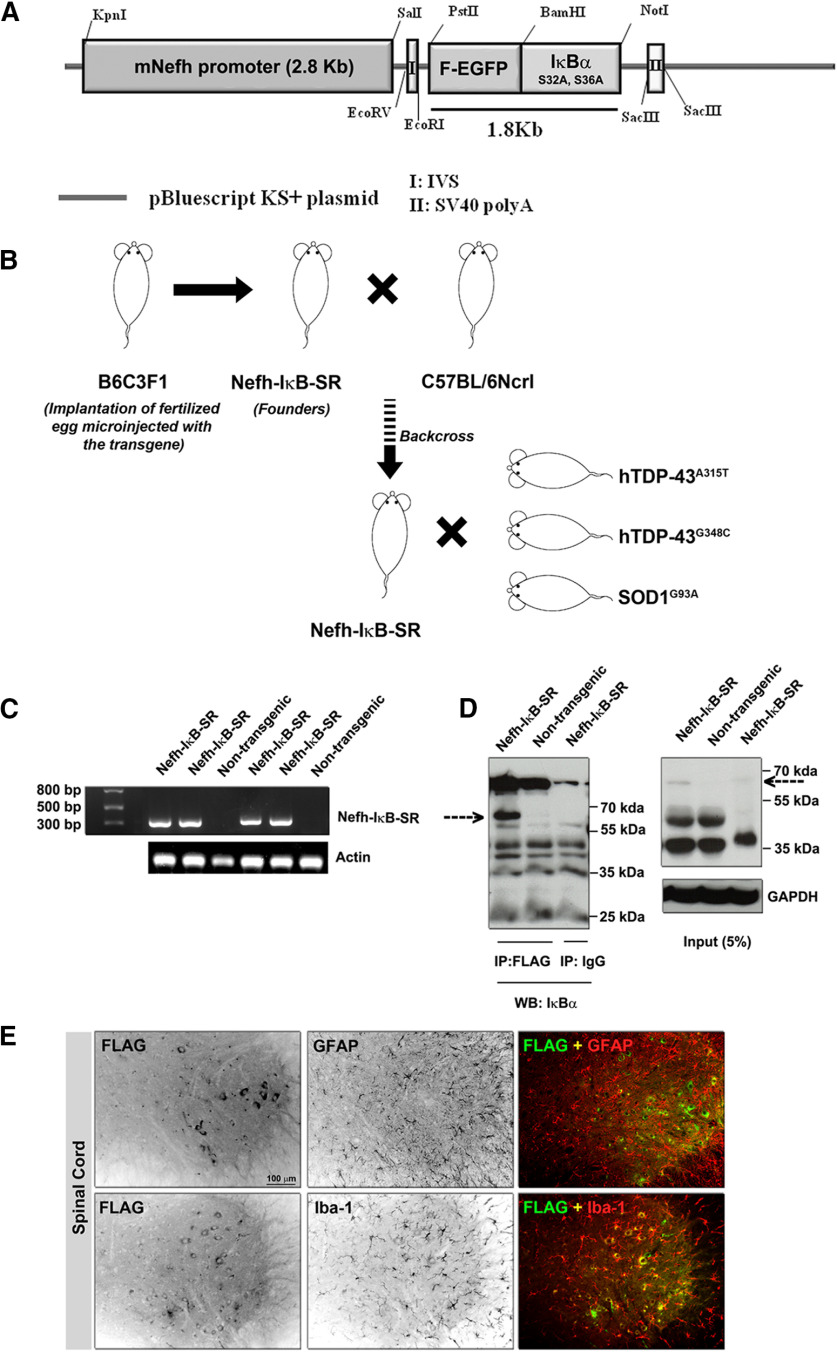
Generation and characterization of the IκB super-repressor mice. The NFH promoter was amplified from mouse genomic DNA and subcloned into the pBluescript KS^+^ backbone. The Flag-EGFp and IκB (SS32, 36AA) fragments were cloned from pEGFP-N3 and PCMV4-3HA/IκBα (SS32, 36AA) plasmids, respectively, and inserted into the pBSKS-Nefh promoter plasmid (***A***). Oocytes that were microinjected with this plasmid were implanted into B6C3F1 mouse to generate the Nefh-IκB^SS32,36AA^ mouse (also known as the IκB super-repressor [SR] mouse). The founders were backcrossed with C57BL/6Ncrl mice before cross-breeding with ALS mouse models (viz TDP-43^A315T^, TDP-43^G348C^, and SOD1^G93A^) (***B***). Genotyping of IκB-SR mice was performed to check a 329 bp fragment from the EGFP gene, which was absent in nontransgenic littermates (***C***). Spinal cord lysates from littermate IκB-SR and WT mice were immunoprecipitated with Flag-M2 antibody and probed with IκBα antibody. Data are a representative image of three separate experiments. Results clearly showed a prominent band at ∼63 kDa corresponding to the combined molecular weight of FLAG-EGFP and IκBα (dotted arrow), which was absent in the WT. This band could not be detected when the Flag-M2 antibody was replaced with normal mouse IgG (***D***). To determine that the transgene was not expressed in other non-neuronal cell types, spinal cord sections were costained for Flag and Iba-1 (microglia marker) or Flag and GFAP (astrocyte marker). Flag expression could not be detected from either Iba-1-positive or GFAP-positive cells (***E***); Extended data Figure 1-1.

Using genetic engineering, we generated a cDNA construct encoding in-frame FLAG, EGFP, and human mutant IκBα^S32A, S36A^ under the control of neurofilament Nefh gene promoter. The Nefh-FLAG/EGFP/IκBα^S32A, S36A^ (Nefh-IκB-SR) DNA construct was used to generate transgenic mice by the microinjection of DNA into fertilized eggs followed by implantation in pseudo-pregnant female mice (Fig. 1*A*). Four transgenic founders were initially generated (#1397, #1398, #1399, #2373), which were then assessed for levels of transgene expression based on FLAG and GFP detection from spinal cord and brain samples. Microscopy of immunostained spinal cord sections revealed the highest neuronal expression levels of FLAG and GFP in founder #1397 (Extended Data Fig. 1-1*A*). Immunoblots of spinal cord lysates confirmed prominent detection of the IκB-SR protein of ∼63 kDa (IκBα + FLAG + GFP) in spinal and brain samples from #1397 transgenic mouse (Extended Data Fig. 1-1*B*,*C*). As the transgene was under the Nefh gene promoter, its expression was limited to the nervous tissues. To confirm this, non-neuronal tissues (kidney, liver, and spleen) were excised from IκB-SR transgenic mice, and their lysates were subjected to SDS-PAGE followed by immunodetection with anti-FLAG or anti-GFP antibodies. Immunoblots revealed no signal in non-neuronal tissues for IκB-SR protein at expected 63 kDa (Extended Data Fig. 1-1*D*). Based on these results, a mouse colony from the founder #1397 was generated by backcrossing transgenic mice in the C57BL6 background. Mice from the IκB-SR line (#1397) were then bred with transgenic models of ALS expressing either mutant TDP-43 species or mutant SOD1^G93A^ (Fig. 1*B*).

10.1523/JNEUROSCI.0536-20.2020.f1-1 Figure 1-1 **Characterization of the IκB-SR mice** Spinal cord sections from the 4 founder mice lines, along with non-transgenic littermate, were sectioned and stained with anti-FLAG antibody. Strongest signal was detected from founder line 1397, whose sections were further stained with anti-GFP antibody to confirm presence of transgene. Magnification=20X; scale bar= 100 μm (A). Spinal cord lysates were also immunoblotted and probed with anti-FLAG and anti-GFP antibodies. The protein expressed by the transgene (approximately 63 kDa; denoted by dotted arrow) could be detected in some founders but never in WT (non-transgenic) mice (B). Similar results were obtained from brain lysates. Data is representative of at least 3 mice per group (C). Kidney, liver and spleen from 3 littermate mice from founder line 1397 were lysed and probed for FLAG and GFP. Lack of any specific signal confirmed that the expression of transgene was limited to nervous tissue (D). IκB-SR and WT mice were intraperitoneally challenged with E. coli LPS to induce inflammation and sacrificed after 12h. Spinal cord and spleen were excised and lysed for immunoblotting. When probed for phospho IκBα and total IκBα, transgenic mice showed no induction in spinal cord but robust induction in spleen. However, IκBα was found to be induced in both spinal cord as well as spleen in WT mice (E). Download Figure 1-1, TIF file

The presence of IκB-SR transgene was confirmed at every step by genotyping (Fig. 1*C*). Spinal cord lysates from IκB-SR and littermate WT mice were immunoprecipitated with anti-FLAG M2 antibody and subsequently fractionated by SDS-PAGE with ensuing immunoblotting with anti-IκBα antibody. A parallel experiment using mouse IgG served as control. Results further confirmed the immunodetection of IκB-SR protein by the anti-IκBα antibody in spinal cord lysates from transgenics but not from WT mice (Fig. 1*D*). To confirm the cell specificity of the transgene expression, spinal cord sections from IκB-SR mice were immunostained for FLAG and GFAP (astrocyte marker) or FLAG and Iba-1 (microglia marker). Results clearly show that none of the GFAP- or Iba-1-positive cells was positive for FLAG, thereby confirming neuron-specific expression of IκB-SR (Fig. 1*E*).

To validate the efficacy of IκB-SR transgene expression to inhibit IκBα activation *in vivo* in the CNS, mice were injected with LPS from *Escherichia coli* intraperitoneally; and after 12 h, the mice were killed for immunodetection of phosphorylated IκBα species in their spinal cord and spleen.

In normal WT mice, LPS treatment led via activation of TLR4 pathway to substantial induction of IκBα phosphorylation in both the spleen and spinal cord (Extended Data Fig. 1-1*E*). In contrast, LPS treatment of IκB-SR transgenic mice caused induction of IκBα phosphorylation in the spleen but not significantly in the spinal cord due to expression of the NF-κB inhibitor construct IκB-SR (Extended Data Fig. 1-1*E*). Moreover, *in vitro* transfection studies with NSC-34 (motor neuron-like) cells confirmed that expression of IκB-SR attenuated levels of phosphorylated IκBα species before or after stimulation by TFN-α (Extended Data Fig. 2-2*A*).

### Reduced nuclear translocation of p65NF-κB in spinal neurons of IκB-SR;TDP-43^A315T^ mice

**Figure 2.**
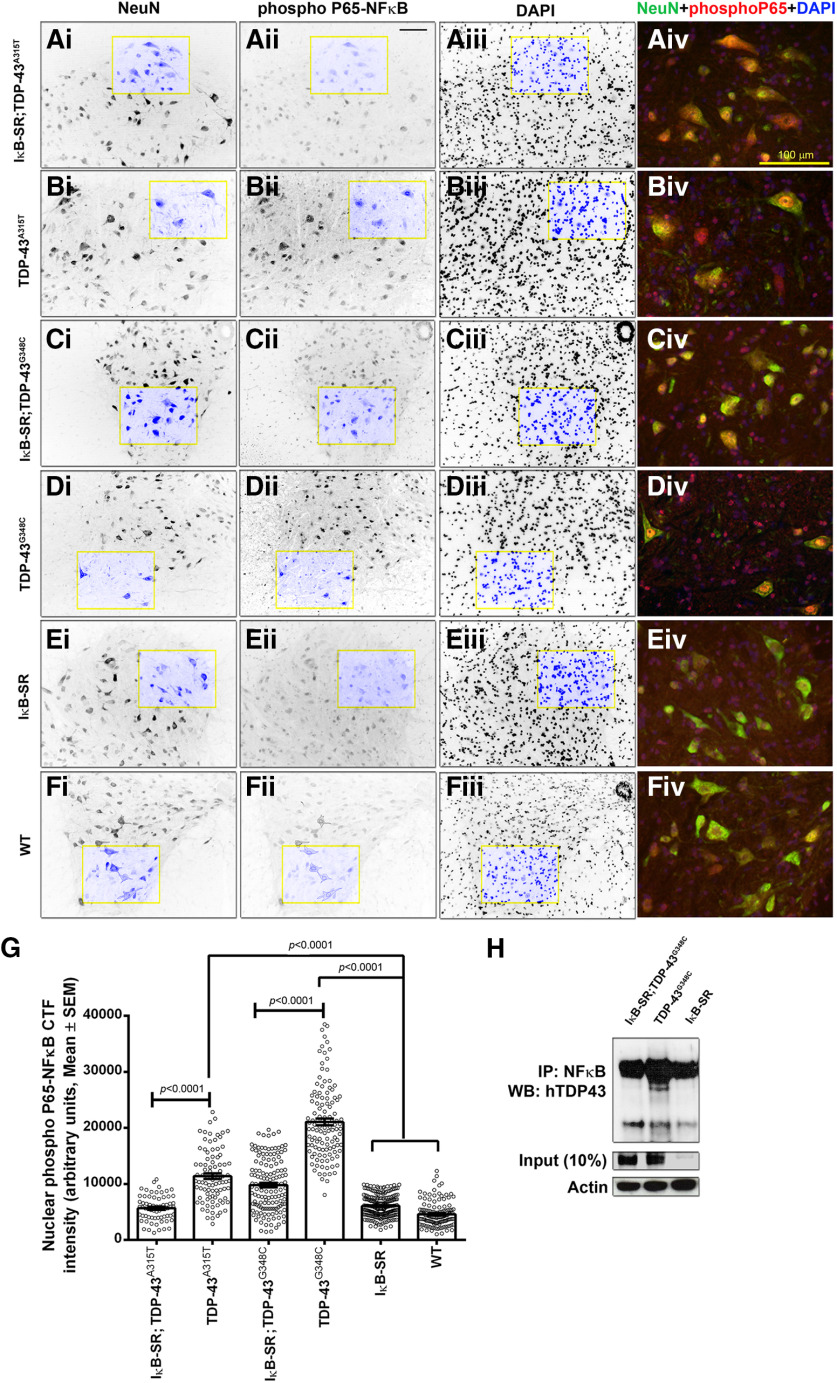
Phospho-P65-NF-κB expression in spinal cord neurons and NF-κB-TDP-43 interaction. To assess the level of inhibition of p65-NF-κB activation, achieved due to the super-repression, and compare them with that in ALS mice models, spinal cord sections were stained with neuron-specific NeuN antibody and phopho-p65 NF-κB antibody. Nuclei were stained with DAPI. Single-channel images from IκB-SR;TDP-43^A315T^ (***Ai-Aiii***), TDP-43^A315T^ (***Bi-Biii***), IκB-SR;TDP-43^G348C^ (***Ci-Ciii***), TDP-43^G348C^ (***Di-Diii***), IκB-SR (***Ei-Eiii***), and WT (***Fi-Fiii***) mice were analyzed separately using Fiji in a three step process. First, three corresponding single-channel images (NeuN, p65-NF-κB, DAPI) of every field were opened and stacked together. At the second step, neuronal boundaries were drawn (based on NeuN staining) using the free-hand selection tool, and the area was measured. Only neurons with an area > 250 µm^2^ were considered for this study. The nuclear boundary was also marked similarly (based on DAPI). Finally, the p65-NF-κB signal in the area marked as nuclear was measured and corrected for background fluorescence. For representational purposes, all channels in ***F*** are showing original boundary markings. Neurons from at least six sections per mouse with 3 mice in each group were considered. Original magnification ×20. The highlighted sections in each single channel images were cropped using Adobe Photoshop CS5, enlarged 300-fold, merged, and displayed in actual colors (***Aiv***, ***Biv***, ***Civ***, ***Div***, ***Eiv***, ***Fiv***). Scale bar, 100 µm. Data were tabulated and the mean ± SEM plotted. ANOVA was performed using Kruskal-Wallis test followed by comparison between groups using Dunn's post-test. ***G***, Adjusted *p* values. The neuronal nuclear phospho-p65 NF-κB expression was found to be significantly elevated in both the ALS mice model compared with IκB-SR and WT. Neurons in the spinal cord of double-transgenic mice (IκB-SR;TDP-43 mutants) showed marked reduction in presence of phospho-p65 NF-κB compared with respective TDP-43 mutants only. To assess whether the inhibition of p65-NF-κB release from the inhibitory κB complex affected its binding with TDP-43, spinal cord lysates from double-transgenic mice (IκB-SR;TDP-43 mutant) and its littermate TDP-43 mutant and IκB-SR were immunoprecipitated with anti-P65-NF-κB antibody and probed with anti-hTDP-43 antibody. Results showed a clear reduction in binding between the two proteins due to IκBα super-repression (dotted arrow). No interaction was expectedly observed in IκB-SR mice, which lacked the human TDP-43 expression. ***H***, Representative of three independent experiments; Extended data Figure 2-2.

To assess the suppression of NF-κB activation in spinal neurons of IκB-SR;TDP-43^A315T^ mice, spinal cord sections were immunostained with anti-phospho p65 NF-κB antibody and counterstained with anti-neuron specific antibody (NeuN) and DAPI to mark the nucleus (Fig. 2*A–F*). Using ImageJ software, the neuronal nuclear area was outlined at first, followed by estimation of phospho-NF-κB signal intensity within that specified region. It is noteworthy that the nuclear signal intensity for phospho-NF-κB was increased in TDP-43^A315T^ mice and TDP-43^G348C^ mice by ∼90% and ∼200%, respectively, compared with WT or IκB-SR mice (Fig. 2*G*). A significant reduction of ∼50% in the nuclear signal of phospho-NF-κB in the neuronal nuclei of IκB-SR;TDP-43^A315T^ mice compared with single-transgenic TDP-43^A315T^mice. An almost equivalent reduction (∼53%) was also measured in IκB-SR;TDP-43^G348C^ mice compared with single-transgenic TDP-43^G348C^. Coimmunoprecipitation studies were performed to evaluate the interaction between NF-κB and TDP-43. Spinal cord lysates from TDP-43 mutant mice or from the respective double-transgenics with IκB-SR were subjected to immunoprecipitation with anti-NF-κB antibody, proteins fractionated by SDS-PAGE with ensuing immunoblotting with the use of antihuman TDP-43 antibody. Results revealed a coimmunoprecipitation of TDP-43 with anti-NF-κB antibody using spinal cord lysate from in TDP-43^A315T^ mice. In contrast, inhibition of NF-κB signaling by IκB-SR prevented interaction of TDP-43 with NF-κB. Thus, no TDP-43 signal was detected in immunoprecipitates from IκB-SR;TDP-43 mice (Fig. 2*H*).

10.1523/JNEUROSCI.0536-20.2020.f2-2 Figure 2-2 The IκB-SR plasmid was cloned into a pcDNA3 backbone. Mouse motor neuron-like NSC34 cells were transfected with this plasmid, with or without exposing the cells to recombinant murine TNF-α (10ng/mL). The levels of phospho NF-κB and total NF-κB were estimated from the cell lysates. In un-transfected cells, TNF-α treatment resulted in increased phosphorylation of NF-κB which was not observed in case of cells that were transfected with the IκB-SR plasmid (A). A 40X magnified image of spinal cord neuron, stained for NeuN and hTDP-43 with borders (cellular and nuclear) drawn around it prior to measurement of nuclear and cytosolic signal intensities (B). Body weights of all male and female mice from the six groups showing no significant intergroup variation which could affect performance in motor performance test (C). Download Figure 2-2, TIF file

### Suppression of NF-κB signaling alleviates cytoplasmic accumulation of TDP-43 in spinal motor neurons

**Figure 3.**
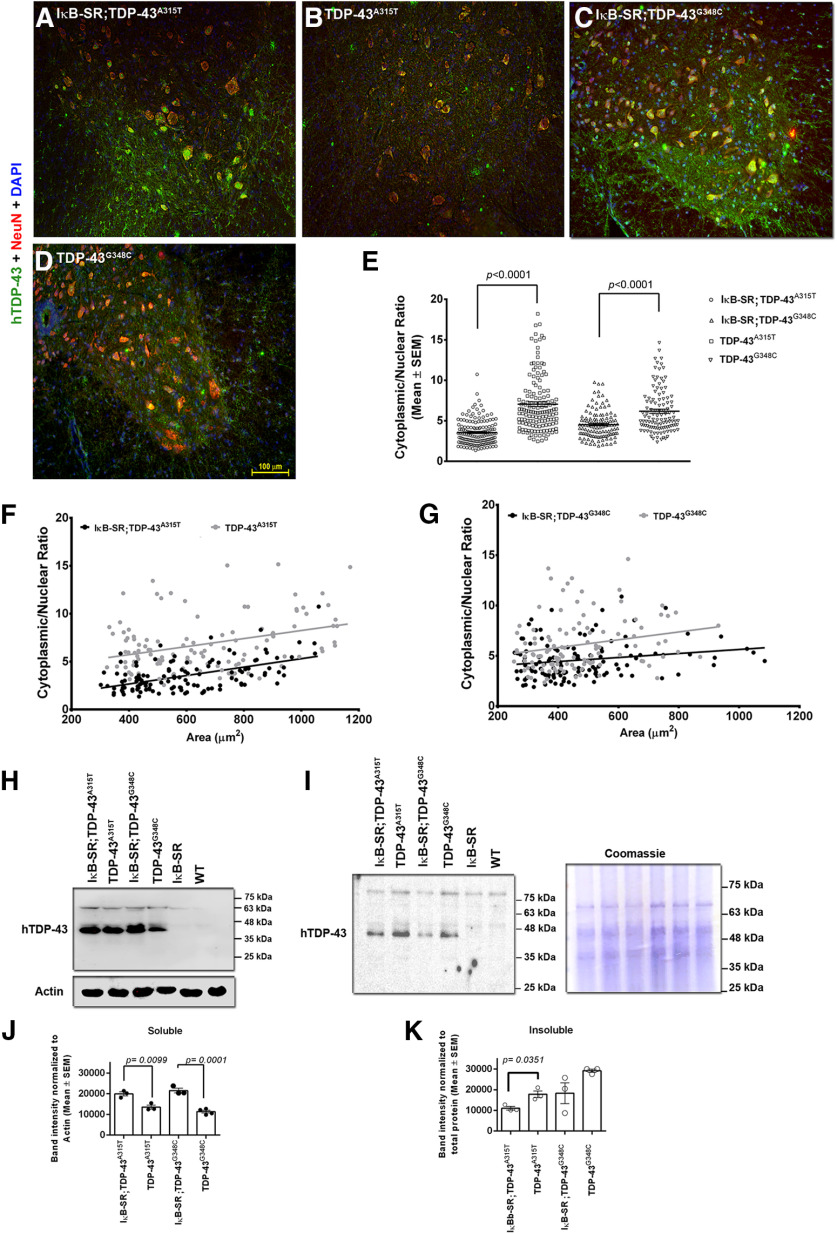
TDP-43 expression in spinal cord neurons. To evaluate the nuclear-cytoplasmic distribution of hTDP-43 in spinal cord neurons of IκB-SR;TDP-43^A315T^, TDP-43^A315T^, IκB-SR;TDP-43^G348C^, and TDP-43^G348C^ mice, sections were stained with anti-human TDP-43 and anti-NeuN antibodies; nuclei were stained with DAPI (***A-D***). Original magnification ×20. Boundaries (cellular and nuclear) were outlined using Fiji, and TDP-43 signal intensity was measured only from neurons with area > 250 µm^2^. The nuclear signal intensity value was subtracted from the signal intensity of whole cell to generate the cytoplasmic signal intensity. The C:N signal intensity was calculated and tabulated. Neurons from at least six sections per mouse with 3 mice in each group were considered. A higher C:N ratio indicated predominantly cytoplasmic localization of TDP-43. ANOVA was performed using Kruskal-Wallis test followed by comparison between groups using Dunn's post-test. Adjusted *p* values are denoted in the figure. Results showed that TDP-43 was predominantly cytoplasmic in neurons in spinal cord of TDP-43 mutant mice. In comparison, there was a significant reduction in the C:N ratio of TDP-43 in spinal neurons of the double-transgenic mice. The C:N did not significantly differ between either the two TDP-43 mutant groups or the two double-transgenic groups (***E***). The hTDP-43 C:N ratio in both IκB-SR;TDP-43^A315T^ and TDP-43^A315T^ groups correlated with increasing neuronal soma size as calculated by Spearman's rank correlation test (95% CI; ρ for Area vs C:N of IκB-SR;TDP-43^A315T^ = 0.4520; *p* < 0.0001; ρ for Area vs C:N of TDP-43^A315T^ = 0.2697; *p* = 0.0033) (***F***). The hTDP-43 C:N ratio in IκB-SR;TDP-43^G348C^ did not significantly correlate with the neuronal soma area, although that in TDP-43^G348C^ did correlate with area (ρ for Area vs C:N of IκB-SR;TDP-43^G348C^ = 0.1677; *p* = 0.0575; ρ for Area vs C:N of TDP-43^G348C^ = 0.2519; *p* = 0.0079) (***G***). Immunoblotting from RIPA-soluble (***H***) and -insoluble (***I***) fractions of spinal cords of mice from various groups showed that in both IκB-SR;TDP-43^A315T^ and IκB-SR;TDP-43^G348C^ mice spinal cords, human TDP-43 was present at a significantly higher level compared with respective TDP-43 mutant mice tissues (***J***). Contrarily, in the RIPA-insoluble fractions, more human TDP-43 was detected from the TDP-43 mutant mice tissues (***K***). Data were analyzed by unpaired Student's *t* test with Welch's correction. *p* values are denoted in the figure.

Transgenic mice expressing human TDP-43 mutants exhibit age-dependent accumulations of human TP-43 in the cytoplasm of spinal motor neurons. Thus, in 1-year-old single-transgenic mice expressing either TDP-43^A315T^ or TDP-43^G348C^, the antihuman TDP-43 immunostaining was mainly detected in the cytoplasm of neurons in the ventral region of the spinal cord (Fig. 3*B*,*D*). In contrast, human TDP-43 was predominantly localized in the nucleus of spinal neurons in sections from double-transgenic mice, either the IκB-SR;TDP-43^A315T^ or IκB-SR;TDP-43^G348C^ mice (Fig. 3*A*,*C*). The TDP-43 signal intensity in cytoplasm and nucleus was estimated by marking the cell and nuclear boundaries (cytoplasm = whole cell – nucleus) using multiple spinal cord sections pooled from 3 mice per group (Extended Data Fig. 2-2*B*). Results revealed a 2-fold decrease in cytoplasmic-to-nuclear (C:N) TDP-43 signal ratio in IκB-SR;TDP-43^A315T^ mice compared with single-transgenic TDP-43^A315T^mice (Fig. 3*E*). There was also a 1.4-fold decrease in C:N signal ratio in IκB-SR;TDP-43^G348C^ compared with TDP-43^G348C^ mice. To assess whether the cytoplasmic accumulation of TDP-43 is affected by the size of the neuron, we tabulated the C:N ratio with the corresponding area of neurons (with cutoff area > 250 µm^2^). The ratios in both IκB-SR;TDP-43^A315T^ and TDP-43^A315T^ groups correlated with increasing neuronal soma size, as calculated by Spearman's rank correlation test (95% CI; ρ for Area vs C:N of IκB-SR;TDP-43^A315T^ = 0.4520; *p* < 0.0001; ρ for Area vs C:N of TDP-43^A315T^ = 0.2697; *p* = 0.0033) (Fig. 3*F*). However, the C:N ratio of TDP-43 signal in IκB-SR;TDP-43^G348C^ did not significantly correlate with the neuronal soma area, although that in TDP-43^G348C^ did correlate with area (ρ for Area vs C:N of IκB-SR;TDP-43^G348C^ = 0.1677; *p* = 0.0575; ρ for Area vs C:N of TDP-43^G348C^ = 0.2519; *p* = 0.0079) (Fig. 3*G*). RIPA-soluble and -insoluble fractions of spinal cords of mice from various groups were subjected to immunoblotting. Results showed that in RIPA-soluble fractions of both IκB-SR;TDP-43^A315T^ and IκB-SR;TDP-43^G348C^ mice spinal cords, human TDP-43 was present at a significantly higher level compared with respective TDP-43 mutant mice tissues (Fig. 3*H*,*J*). Contrarily, in the RIPA-insoluble fractions, more human TDP-43 was detected from the TDP-43 mutant mice tissues (Fig. 3*I*,*K*).

### Neuronal IκB-SR expression conferred protection of motor neurons with ensuing improvement of motor performance

**Figure 4.**
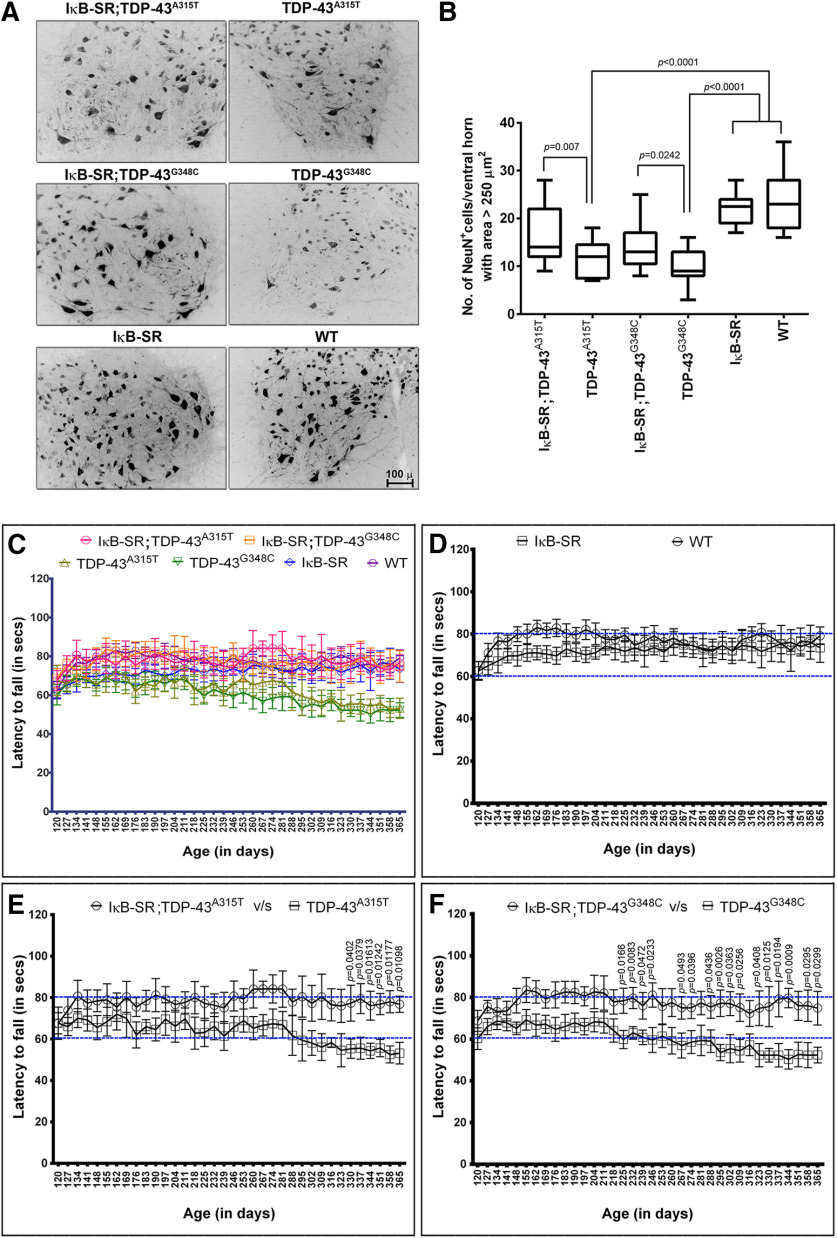
Motor activities of the transgenic mice. To assess the effect of NF-κB activation inhibition on motor neuron count in IκB-SR;TDP-43^A315T^ and IκB-SR;TDP-43^G348C^ mice and compare them with that in age-matched TDP-43^A315T^, TDP-43^G348C^, IκB-SR, and WT littermates, spinal cord sections were stained for the neuronal marker NeuN (***A***). Original magnification ×20. Scale bar, 100 µm. The number of NeuN-positive cells in ventral horn with an area > 250 µm^2^ were counted using Fiji, and data were represented as a box-and-whisker graph showing minimum to maximum range of values. Data were analyzed by one-way ANOVA with Tukey's post-test that clearly showed that 1-year-old IκB-SR;TDP-43 mutant mice had significantly more motor neurons compared with respective TDP-43 mutants. However, these levels were still not at par with those observed in IκB-SR or WT. Data are representative of 3-5 mice per group. ***B***, Adjusted *p* values. To determine whether the spinal motor neuron count translated to motor performance, the mice in all six groups were trained to run on an accelerating rotarod from 4 months of age. Data were recorded weekly until 12 months of age and represented as the mean of maximum latency to fall from the rotarod at each time point. *N* = 10-15 per group (***C***). Comparison of rotarod performance of WT and IκB-SR mice does not show any significant difference over the duration of the protocol (4-12 months) (***D***). Comparison of motor performances of mice belonging to IκB-SR;TDP-43^A315T^ and TDP-43^A315T^ groups revealed an initiation of decline in the TDP-43 mutant mice at ∼280 d of age. However, because of the variance in results, this became statistically significant difference beyond 330 d of age. In contrast, the IκB-SR;TDP-43^A315T^ mice maintained their performance at a steady level. Data were analyzed by multiple *t* test, one per row (***E***). A similar pattern of decline was observed while comparing between IκB-SR;TDP-43^G348C^ and TDP-43^G348C^. However, in TDP-43^G348C^ mice, the decline was initiated at an earlier time point (225 d), and their motor performance at this age was significantly different from that of IκB-SR;TDP-43^G348C^ mice (***F***). Data were analyzed by multiple *t* test, one per row, and the *p* values are denoted in the figure.

Studies have shown that α motor neurons are vulnerable in ALS, whereas γ motor neurons remain mostly unaffected (Conradi and Ronnevi, 1993; Lalancette-Hebert et al., 2016). For estimation of α motor neuron numbers in spinal ventral horns, a size-based approach was adopted in the current study, rather than detection with the classical marker ChAT. ChAT is expressed in both α and γ motor neurons, whereas the neuronal DNA-binding protein NeuN is expressed in α but not (or poorly) in γ motor neurons (Friese et al., 2009; Shneider et al., 2009; Gusel'nikova and Korzhevskiy, 2015). γ motor neuron cell body area ranges from 100 to 250 μm^2^, whereas the larger α motor neurons range from 250 to 1200 μm^2^ (Drachman and Rothstein, 2000; Pasetto et al., 2017). In our study, we have considered NeuN-positive cells in spinal ventral horn with area >250 μm^2^ as α motor neurons. The cells were from multiple spinal cord sections chosen at random from at least 3 mice per group (Fig. 4*A*). Results showed that there were significantly more α motor neurons in spinal cord of IκB-SR;TDP-43^A315T^ mice compared with TDP-43^A315T^ mice (17.24 ± 1.573 vs 11.47 ± 0.875). A similar difference was also observed between IκB-SR;TDP-43^G348C^ and TDP-43^G348C^ (14.29 ± 1.007 vs 9.783 ± 0.7724). Nonetheless, these values were still significantly lower than that recorded either from IκB-SR (21.82 ± 0.613) or WT (23.52 ± 1.228) mice (Fig. 4*B*), suggesting that IκB-SR transgene expression did not completely rescue the loss of α motor neurons in the TDP-43^A315T^ and TDP-43^G348C^ mice.

The motor phenotypes in these TDP-43 ALS mouse models are characterized by the progressive loss of neuromuscular junctions and decrease of motor performance (Swarup et al., 2011a). To assess the effect of chronic inhibition of neuronal NF-κB signaling on motor performance, the mice from all six genotypes were trained to run on an accelerating rotarod from the age of 120 d (4 months) and monitored weekly until 12 months of age. The maximum latency to fall from the rotarod out of three trials per mouse per week was recorded and tabulated together (Fig. 4*C*). To elucidate the comparisons and ease of analysis, the data were split to show IκB-SR versus WT, IκB-SR;TDP-43^A315T^ versus TDP-43^A315T^, and IκB-SR;TDP-43^G348C^ versus TDP-43^G348C^ groups. There was no significant difference in motor performance of IκB-SR transgenic mice compared with their nontransgenic littermates, with latency to fall of ∼80 s (Fig. 4*D*). In contrast, during aging (starting at ∼300 d of age), the TDP-43^A315T^ mice and TDP-43^G348C^ mice exhibited decline of motor performance in the rotarod test with latency to fall within ∼60 s (Fig. 4*C*,*E*,*F*). Remarkably, neuronal expression IκB-SR in double-transgenic mice rescued the age-dependent deficits of motor performance due to expression of TDP-43 mutations. Hence, the double-transgenic IκB-SR;TDP-43^A315T^ and IκB-SR;TDP-43^G348C^ mice exhibited latency to fall of ∼80 s in the rotarod test. There were no significant differences in the body weights of mice from all six groups, which could affect fluctuations in the rotarod performance (Extended Data Fig. 2-2*C*).

### Neuronal inhibition of NF-κB signaling mitigated cognitive deficits in transgenic mice expressing ALS-linked TDP-43 mutations

**Figure 5.**
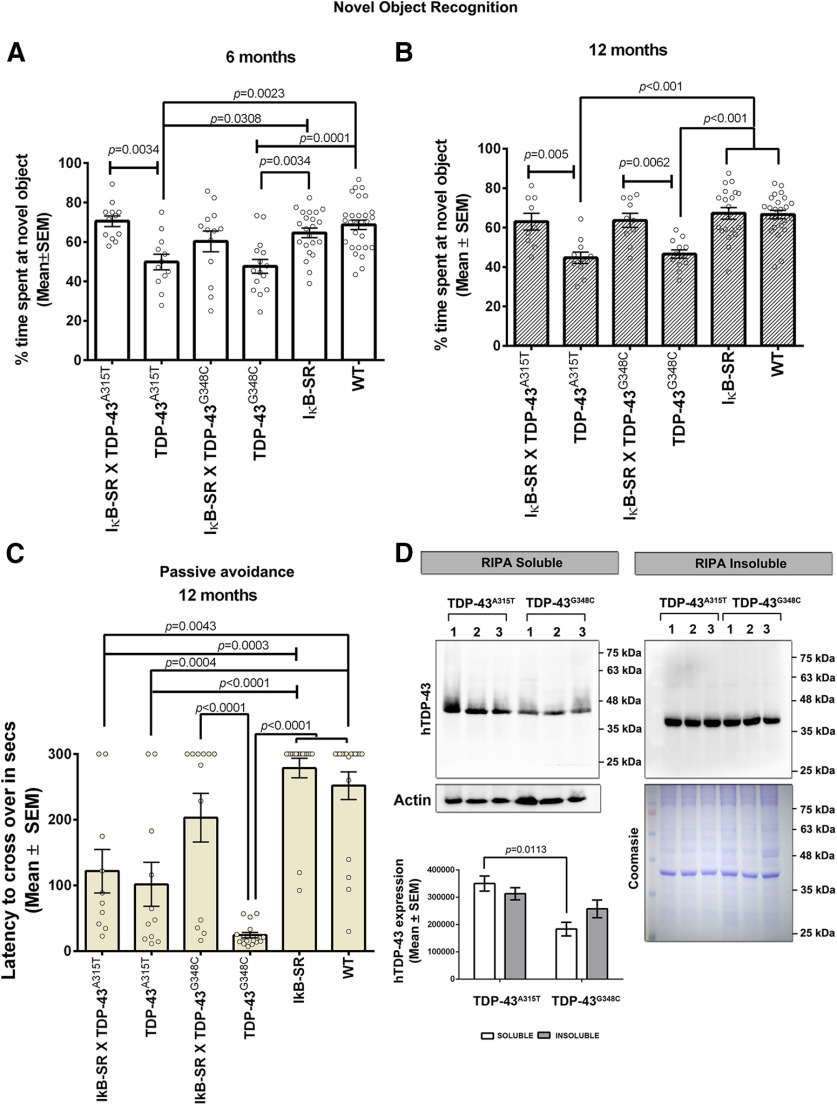
Determination of cognitive abilities in TDP-43 mutant and IκB super-repressor mice. Novel object recognition test performed at 6 months (***A***) and 12 months (***B***) of age showed mice expressing mutant TDP-43 with impaired recognition of the novel object. Contrarily, IκB-SR;TDP-43 mutant mice performed relatively similar to WTor IκB-SR mice. Data are representative of independent mice tested (dots) and represented as mean ± SEM of percent time spent at the novel object. Data were analyzed using one-way ANOVA with Tukey's multiple comparison post-test. Adjusted *p* values are denoted in the figure. ***C***, Passive avoidance test to evaluate fear-induced memory retention indicated cognitive decline in TDP-43^G348C^ mice at 12 months of age, compared with littermate WT and IκB-SR mice. The IκB-SR;TDP-43^G348C^ double-transgenic mice performed significantly better in the memory function test compared with the TDP-43^G348C^ mice. No significant difference was observed between performance of IκB-SR;TDP-43^A315T^ and TDP-43^A315T^ mice, which in either cases was significantly less than littermate WT or IκB-SR mice. Data are representative of independent mice tested (dots) and represented as mean ± SEM of time (in seconds) taken to enter the dark chamber. Data were analyzed using one-way ANOVA with Tukey's multiple comparison post-test. Adjusted *p* values. ***D***, Immunoblot analysis from hippocampal extracts (RIPA-soluble and -insoluble fractions) of three nonlittermate TDP-43^A315T^ and TDP-43^G348C^ mice showed significantly higher TDP-43 expression in TDP-43^A315T^. Data were analyzed by two-way ANOVA followed by Tukey's multiple comparison test. Adjusted *p* values.

In a previous study, we reported age-dependent cognitive declines in transgenic mice expressing ALS-linked TDP-43 mutations (Swarup et al., 2011a). Here, we tested the effects of suppressing neuronal NF-κB signaling in mice and subjected them to the object recognition test at 6 and 12 months of age. The single transgenics TDP-43^A315T^ and TDP-43^G348C^ exhibited impaired performance at novel object recognition with significant reduction in exploration time at novel object compared with their WT or IκB-SR littermates (Fig. 5*A*,*B*). Remarkably, neuronal expression of IκB-SR transgene in mice expressing TDP-43 mutations restored their preference for the novel object at a degree comparable with WT or IκB-SR mouse littermates (Fig. 5*A*,*B*).

At 12 months of age, a passive avoidance test was also performed to assess the fear-induced learning and memory capacities of the animals belonging to each mouse genotype. Results showed that TDP-43^G348C^ mice did not recollect the adverse electric shock experience, and they entered the dark chamber within a few seconds. In contrast, mouse littermates with the IκB-SR;TDP-43^G348C^ genotype performed very well with an average latency of ∼200 s, which is merely below the latency mice belonging to the IκB-SR and WT groups. Somehow, no significant difference in the passive avoidance test was observed between the IκB-SR;TDP-43^A315T^ mice and TDP-43^A315T^ mice (Fig. 5*C*). The performance of these TDP-43^A315T^ groups significantly differed from those of TDP-43^G348C^, IκB-SR, and WT mice. Hippocampal extracts from nonlittermate 12-month-old mice were subjected to immunoblotting to check TDP-43 levels. Results revealed higher levels of TDP-43 in hippocampal extracts from the TDP-43^A315T^ mice than from TDP-43^G348C^ mice. However, the proportion of detergent-insoluble TDP-43 to soluble TDP-43 was more elevated in extracts from TDP-43^G348C^ than in extracts from TDP-43^A315T^ mice, which could explain the discrepancy in the performance at passive avoidance test (Fig. 5*D*).

### Neuronal IκB-SR expression attenuated gliosis

**Figure 6.**
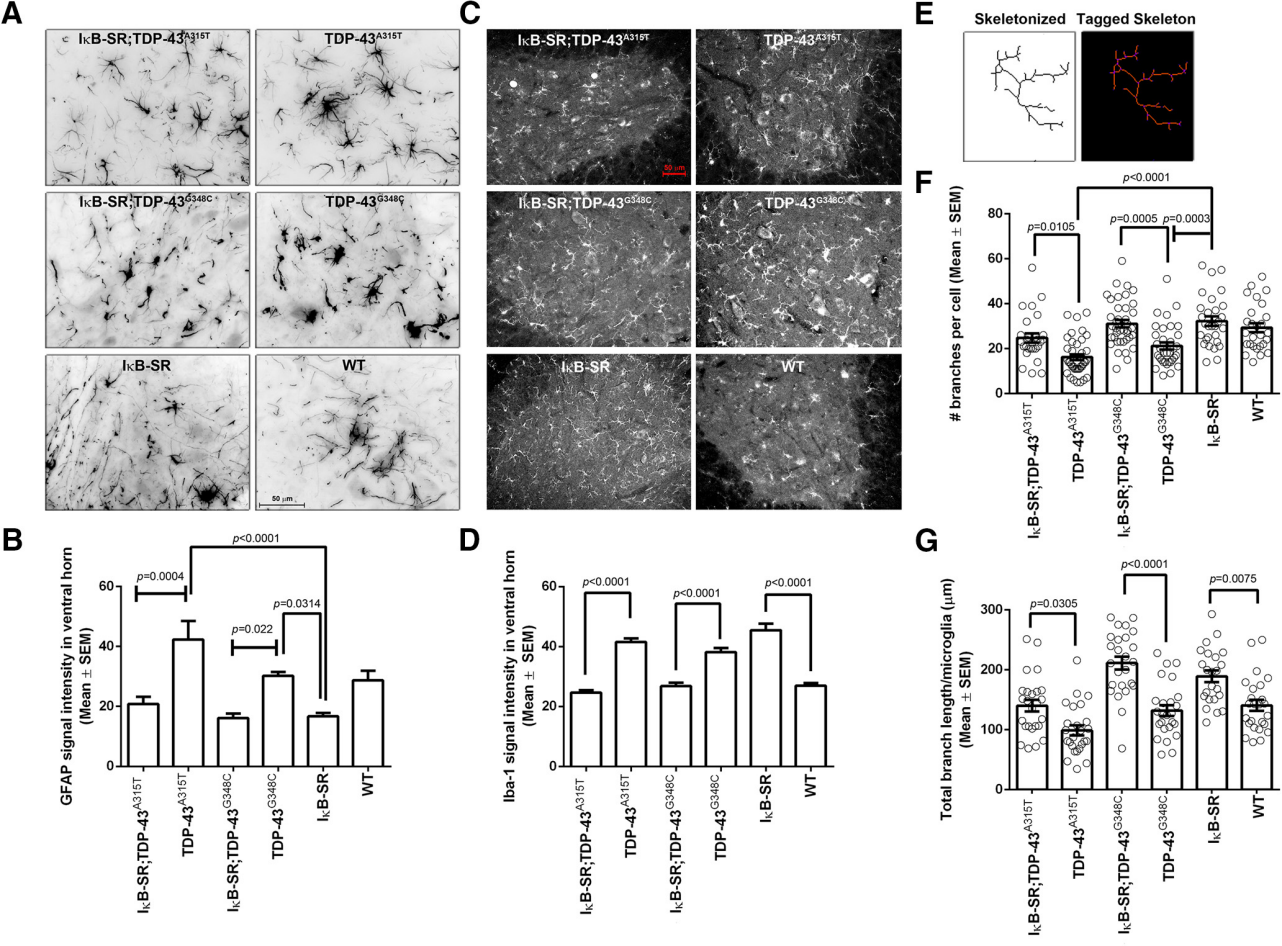
Effect of IκB super-repression on glial activation pattern in spinal cord. Spinal cord sections stained with anti-GFAP antibody to visualize astrocytes in the six groups. Original magnification ×40. Scale bar, 50 µm. Images are representative of 3 mice per group (***A***). The GFAP signal intensity from ventral horn was quantified using Fiji and data represented in tabular form. Results show that GFAP signal intensity to be significantly elevated in spinal cords of TDP-43 mutant mice that could indicate astrogliosis and/or reactive astrocytosis. The signal intensities were significantly lesser in IκB-SR;TDP-43 mutant mice. Data are representative of at least six sections each from 3 mice per group. Data were analyzed by one-way ANOVA along with Tukey's multiple comparison post-test. ***B***, Adjusted *p* values. To visualize microglial morphology, spinal cord sections were stained with anti-Iba1 antibody. Images are representative of 3 mice per group. Original magnification ×20. Scale bar, 50 µm (***C***). The Iba-1 signal intensity from ventral horn was quantified using Fiji and data represented in tabular form. Data show signal intensity to be significantly higher in spinal cords of TDP-43 mutant mice and also IκB-SR mice compared with IκB-SR;TDP-43 mutants or WT mice. Data are representative of at least six sections each from 3 mice per group. Data were analyzed by one-way ANOVA along with Tukey's multiple comparison post-test. ***D***, Adjusted *p* values. Spatiotemporal analysis of microglial morphology was done also with the help of Fiji. The cells were converted to their skeleton form (***E***) followed by analysis of number of branches (processes) per cell (***F***) and total branch (process) length of each cell (***G***). Results show that microglia in spinal cord of TDP-43 mutant mice have significantly reduced number of branches as well as decreased total branch length compared with IκB-SR;TDP-43 mutants or IκB-SR or WT mice, implying a more amoeboid reactive morphology. Each dot represents data from individual neurons. Data are pooled from at least six sections each from 3 mice per group. Data were analyzed by one-way ANOVA along with Tukey's multiple comparison post-test. Adjusted *p* values are denoted in the figure.

To investigate the effects of IκB-SR on neuroinflammation, spinal cord sections from the six groups of mice were immunostained with antibody against GFAP, a marker of astrocytes, and with antibody against Iba1, a marker of microglia (Fig. 6*A*,*C*). Immunofluorescence microscopy revealed a marked upregulation of GFAP signals reflecting astrocytosis in single-transgenic mice expressing TDP-43 mutations compared with those from double-transgenic IκB-SR;TDP-43^A315T^ and IκB-SR;TDP-43^G348C^ mice. The GFAP signal intensities in IκB-SR;TDP-43^A315T^ and IκB-SR;TDP-43^G348C^ mice were similar to the GFAP immunostaining in single IκB-SR transgenic mice (Fig. 6*B*).

The morphology of activated microglia is known to be extremely plastic. Between their resting filamentous or hyper-ramified morphology and phagocytic amoeboid shape, microglia undergoes multiple phenotypic alterations as it responds to the surrounding environment (Kreutzberg, 1996). Since they are finely tuned to neuronal and glial function through continuous intercellular cross-talk and *in vivo* motility, quantitative analysis of microglia morphologies can serve as indicators of neuropathology in CNS (Young and Morrison, 2018). Iba-1 staining was performed on spinal cord sections from the six groups to visualize microglial morphology (Fig. 6*C*). The overall Iba-1 signal intensity was significantly higher in the single-transgenic mice expressing TDP-43 mutations compared with the double-transgenics IκB-SR;TDP-43^A315T^ and IκB-SR;TDP-43^G348C^.

Surprisingly, the Iba-1 signal intensity in spinal cords of IκB-SR mice was also abnormally elevated (Fig. 6*D*). As the microglial morphology distinctly differed in each of the six groups, a spatiotemporal analysis was required to better understand their reactive states. The cell images were converted to their binary skeleton form (Fig. 6*E*), which was then analyzed for branching patterns by processes described in Materials and Methods. The results of this analysis showed that, in both TDP-43^A315T^ and TDP-43^G348C^ mice spinal cords, there was significantly less branching points and decreased branch lengths per microglial cell compared with those observed in either IκB-SR;TDP-43^A315T^ or IκB-SR;TDP-43^G348C^ mice. Thus, the number of branching points and total branch lengths in these double-transgenic mice were similar to that observed in the single IκB-SR mice, indicating that the spinal cord microglia in these groups were more ramified and in a relatively less reactive state (Fig. 6*F*,*G*).

### Inhibition of NF-κB signaling by IκB-SR induced autophagy

**Figure 7.**
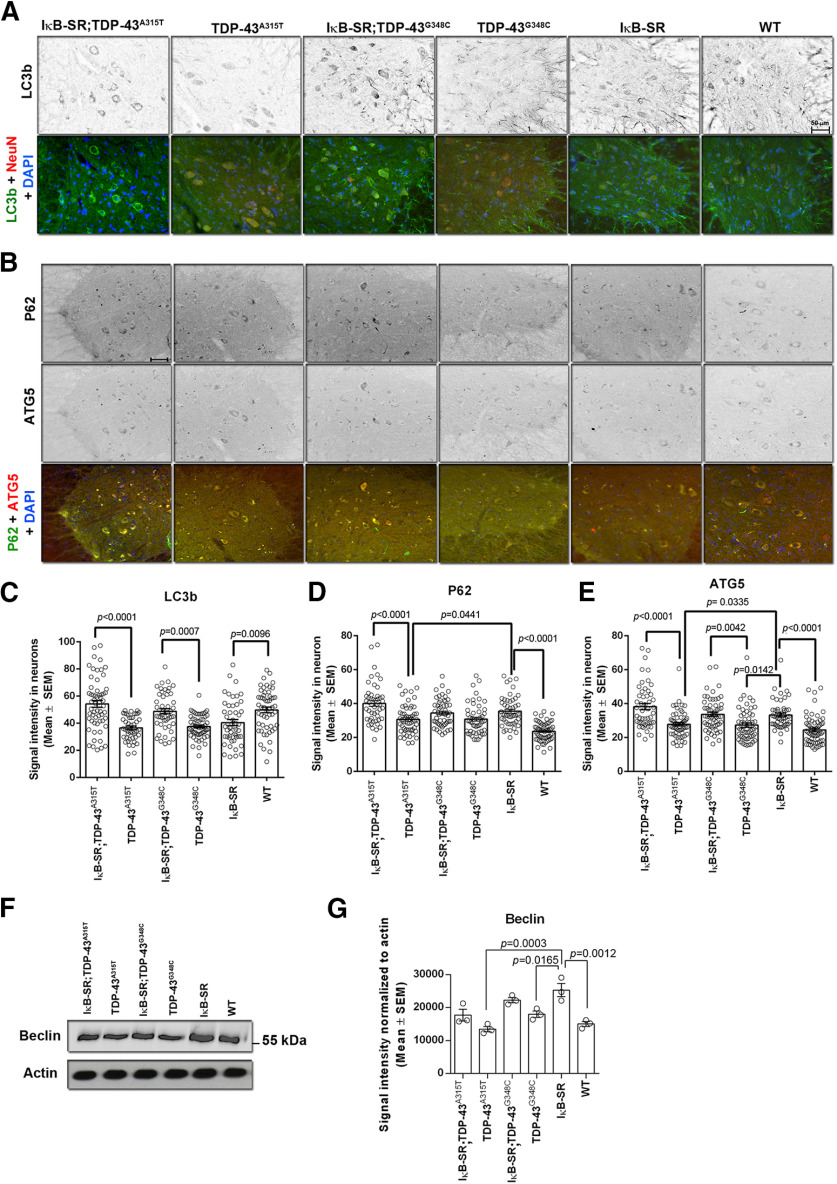
Changes in autophagy markers due to IκB super-repression. Spinal cord sections were stained for different markers of autophagy: LC3B, P62, and ATG5. Images in the figure are representative of 3 mice per group. Original magnification ×20. Scale bars: ***A***, ***B***, 50 µm. The signal intensity of these markers in spinal cord neurons with an area > 250 µm^2^ was measured using Fiji, and the data are represented in tabular form (***C-E***). Results clearly shows significantly reduced expression of autophagy markers in spinal cord neurons of TDP-43 mutant mice compared with IκB-SR;TDP-43 mutants or WT mice. The levels of P62 and ATG5 were also found to be significantly higher in IκB-SR mice compared with WT. Immunoblot performed from spinal cord lysates to detect Beclin showed an increase in the protein expression in IκB-SR mice compared with WT and TDP-43 mutants. The Beclin expression in IκB-SR;TDP-43 mutant mice was slightly elevated compared with only TDP-43 mutants. Data are representative of 3 mice per group. Data were analyzed by one-way ANOVA with Tukey's multiple comparison post-test. ***F***, ***G***, Adjusted *p* values.

Immunofluorescent staining at microscopy for LC3B, P62, and ATG5, markers of autophagy, revealed higher signals in neuronal cytoplasm of spinal cord sections from double-transgenic mice coexpressing IκB-SR with TDP-43 mutations than in sections from single-transgenic TDP-43^A315T^ and TDP-43^G348C^ mice (Fig. 7*A-E*). Moreover, Western blot analysis of spinal cord lysates revealed increased levels of beclin1, another autophagy marker, in transgenic mice expressing the IκB-SR transgene (Fig. 7*F*,*G*).

### Neuronal expression of IκB-SR delayed disease progression and mortality in SOD1^G93A^ mice

**Figure 8.**
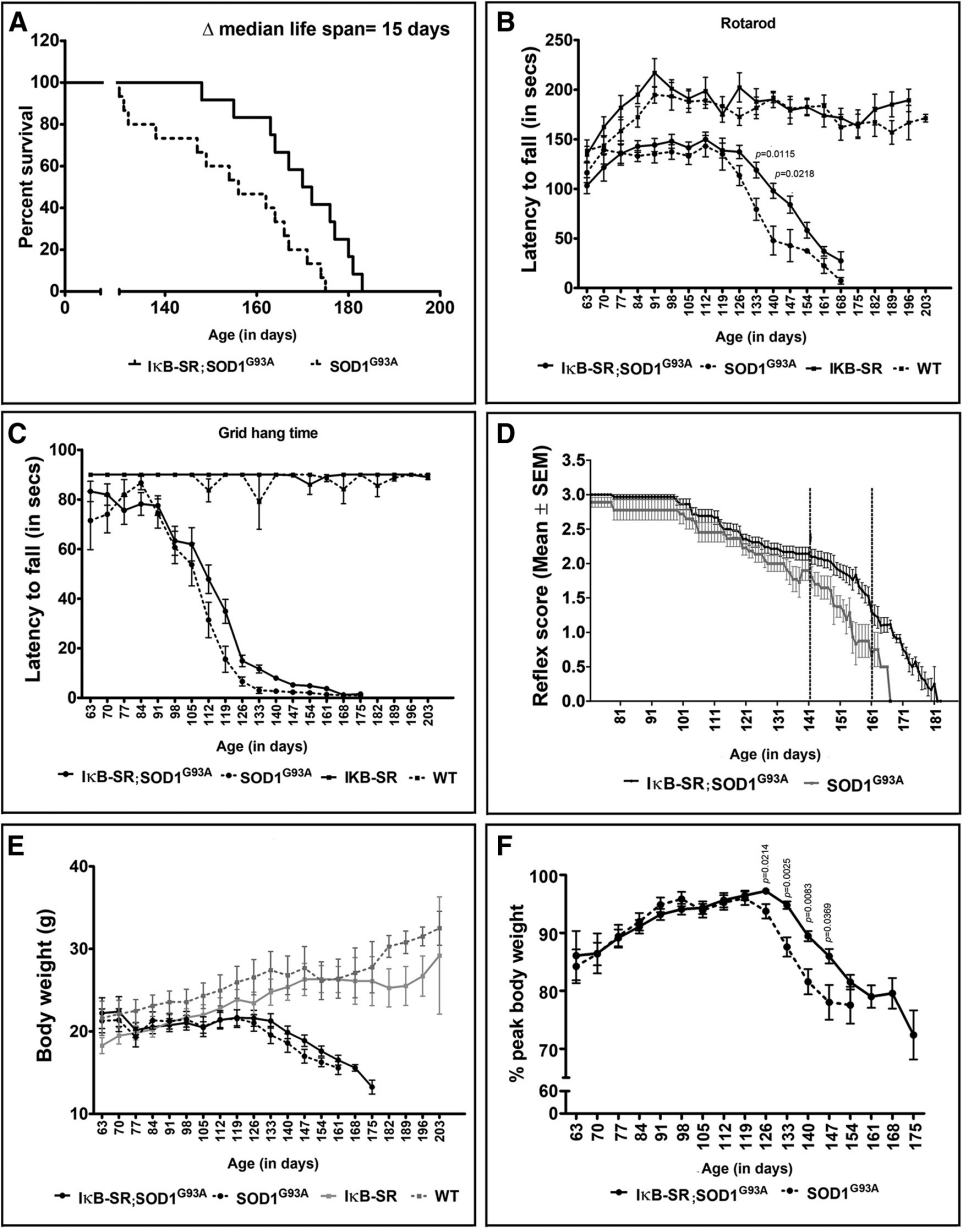
IκB super-repression extends life span of SOD1^G93A^ mice and slows rate of disease progression. On comparing the life span of IκB-SR;SOD1^G93A^ mice with that of only SOD1^G93A^, a median increase of 15 d was observed. Data were analyzed by the Mantel-Cox log rank test; *n* = 25 in each group; *p* = 0.0020 (***A***). Rotarod analysis clearly showed a decline in performance by IκB-SR;SOD1^G93A^ and SOD1^G93A^ relative to that in IκB-SR and WT mice. The performance of SOD1^G93A^ mice was found to differ significantly from that in IκB-SR;SOD1^G93A^ between 133 and 140 d of age. Data were compared by Student's *t* test along with Welch's correction at each time point; the *p* values are denoted in the figure. *N* = 25 in IκB-SR;SOD1^G93A^, 15 in SOD1^G93A^, 8 in IκB-SR, and 10 in WT (***B***). The grid-hang time of the IκB-SR;SOD1^G93A^ and SOD1^G93A^ mice was also clearly worse than IκB-SR and WT mice. However, no significant differences were observed at any time point between IκB-SR;SOD1^G93A^ and SOD1^G93A^ mice; *n* = 25 in IκB-SR;SOD1^G93A^, 15 in SOD1^G93A^, 8 in IκB-SR, and 10 in WT (***C***). Hindlimb reflex scoring showed a slower rate of decline in IκB-SR;SOD1^G93A^ mice compared with SOD1^G93A^ between 141 and 161 d of age (denoted by dotted lines). Data were compared by Student's *t* test along with Welch's correction at each time point, and the *p* values are listed in Extended Data Table 1-1; *n* = 20 in IκB-SR;SOD1^G93A^ and 15 in SOD1^G93A^ (***D***). The actual body weight comparison did not show any difference between IκB-SR;SOD1^G93A^ and SOD1^G93A^ over their life span (***E***). However, on converting the body weights as percentage of peak weight, a significant difference could be observed between 126 and 147 d of age. Data were compared by Student's *t* test along with Welch's correction at each time point. *p* values are denoted in the figure; *n* = 20 in IκB-SR;SOD1^G93A^; 15 in SOD1^G93A^ (***F***).

We have further examined the effects of neuronal suppression of NF-κB in SOD1^G93A^ mice, another model of ALS. As shownin Figure 8*A*, neuronal expression of IκB-SR transgene in SOD1^G93A^ mice resulted in an increase of median survival by 15 d (171 d for IκB-SR;SOD1^G93A^ vs 156 d for SOD1^G93A^). The survival was extended by >20 d in some litters (data not shown).

The locomotor performance of the mice was compared on an accelerating rotarod. The IκB-SR;SOD1^G93A^ and SOD1^G93A^ performed similarly between 63 and 126 d of age. Then there was a sharp decline in latency to fall for the SOD1^G93A^ mice until 146 d of age. The decline in performance was significantly less pronounced with IκB-SR;SOD1^G93A^ between 126 and 146 d of age (Fig. 8*B*). The grid-hang test was performed to assess hindlimb strength, but there was no significant difference between the two mouse groups throughout their life span (Fig. 8*C*). On the other hand, the reflex scores of double-transgenic mice were superior to those of single SOD1^G93A^ mice at end stage of disease starting at 146 d of age (Fig. 8*D*, Extended data Table 1-1). Loss of body weight was also monitored. We found no significant difference between the actual body weights of the mice belonging to either groups (Fig. 8*E*). However, when the data were plotted as percentage of maximum, the body weight of mice belonging to the SOD1^G93A^ group peaked at 119 d of age, whereas for IκB-SR;SOD1^G93A^ it peaked at 126 d. Between 126 and 147 d of age, there was a significant difference between the two groups, indicating a moderate slower rate of disease progression in the IκB-SR × SOD1^G93A^ mice (Fig. 8*F*).

### Reduced levels of misfolded SOD1 in IκB-SR;SOD1^G93A^ mice at different stages of disease

**Figure 9.**
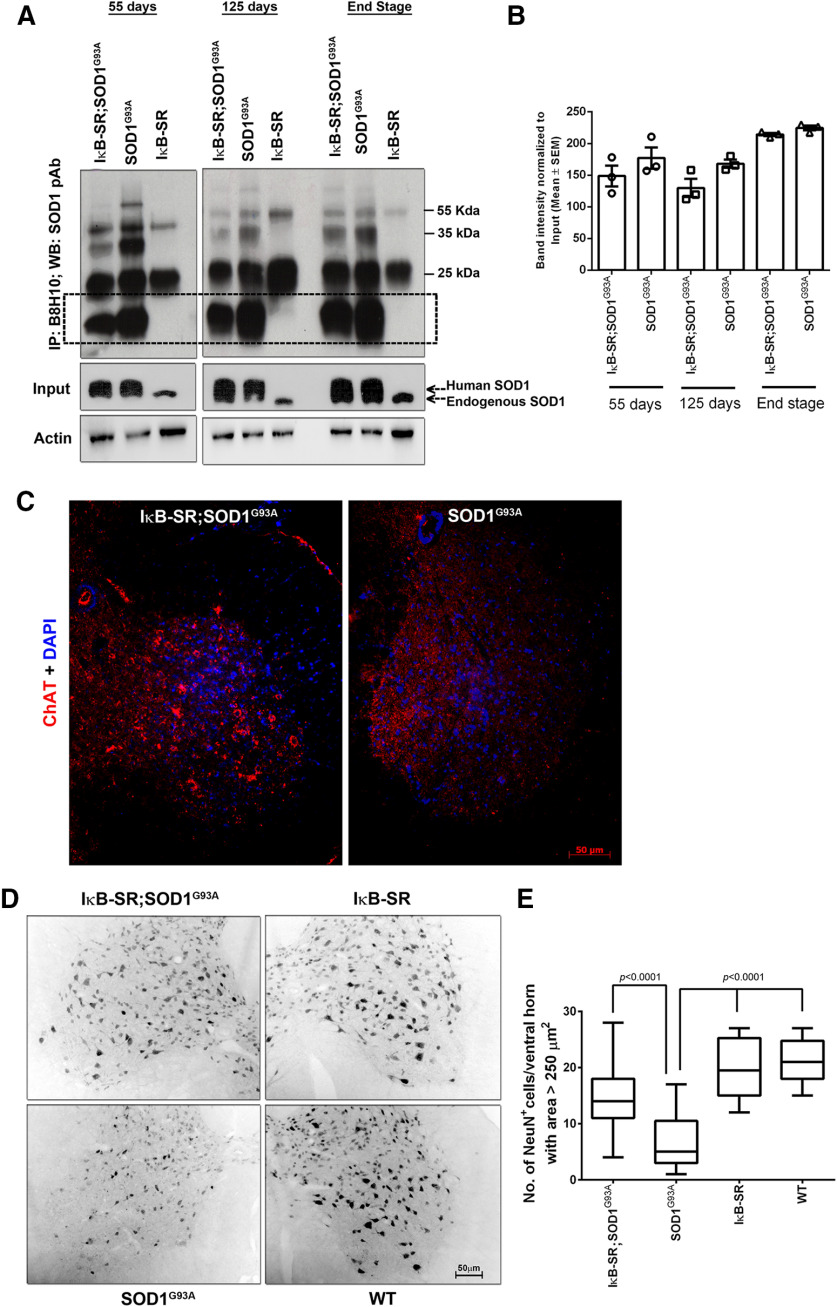
Neuronal IκB super-repression prevents misfolding of SOD1 and extends motor neuron survival in SOD1^G93A^ model. Levels of misfolded SOD1 were detected in spinal cord lysates at presymptomatic, early symptomatic, and end stage by immunoprecipitation. Compared with SOD1^G93A^, IκB-SR;SOD1^G93A^ always presented lesser amounts of misfolded SOD1. Data are representative of 3 mice per group (***A***). Quantitative analysis of the immunoblots also reflects this tread. However, the difference in the levels of misfolded SOD1 between IκB-SR;SOD1^G93A^ and SOD1^G93A^ were not statistically significant (***B***). Spinal cord sections from both IκB-SR;SOD1^G93A^ and SOD1^G93A^ mice were immunostained to detect ChAT. ChAT expression was found to be pronounced in spinal cord of IκB-SR;SOD1^G93A^ mice compared with only SOD1^G93A^ mice. Original magnification ×20. Scale bar, 50 µm. Data are representative of 3 mice per group (***C***). Motor neuron numbers in spinal cord sections were estimated by staining them with NeuN and counting positively stained cells with an area >250 µm^2^. Images are representative of 3 mice per group. Original magnification ×20. Scale bar, 50 µm (***D***). Data were tabulated and represented as box-and-whisker graph showing minimum to maximum range of values. Data were analyzed by one-way ANOVA with Tukey's post-test. ***E***, Adjusted *p* values.

Accumulation of misfolded SOD1 in spinal motor neurons is a pathologic hallmark of mutant SOD1-mediated disease that can be detected in SOD1^G93A^ mouse as early as 30 d of age (Gros-Louis et al., 2010). Using an antibody (B8H10) specific against misfolded SOD1, we examined the levels of misfolded SOD1 in spinal cord extracts by immunoprecipitation with B8H10 antibody followed by PAGE and immunoblotting with anti-pan SOD1 antibody. In all three time points (presymptomatic 55 d, early symptomatic 125 d, and end stage), the levels of misfolded SOD1 were lower (up to ∼40% at 55 d, ∼30% at 125 d, and ∼10% at end stage) in spinal cord samples of IκB-SR;SOD1^G93A^ mice than in samples from age-matched littermate SOD1^G93A^ (Fig. 9*A*). However, together, the differences between IκB-SR;SOD1^G93A^ and SOD1^G93A^ were not statistically significant (Fig. 9*B*). The enzyme ChAT is necessary for maintaining motor neuron functions; and in ALS cases, these cells lose ChAT immunoreactivity before degeneration (Oda et al., 1995). At early symptomatic stage (125 d), spinal cord neurons in IκB-SR;SOD1^G93A^ mice retained ChAT immunoreactivity, unlike SOD1^G93A^ mice (Fig. 9*C*). This result is consistent with the higher number of motor neurons (area > 250 µm^2^) NeuN^+^ detected in the ventral horn spinal cord of IκB-SR;SOD1^G93A^ (14.81 ± 0.7173) compared with SOD1^G93A^ (6.485 ± 0.7883) (Fig. 9*D*,*E*).

## Discussion

Abnormal cytoplasmic aggregates of TDP-43 in degenerating neurons are being recognized as a hallmark of ALS, frontotemporal lobar degeneration (FTLD), and limbic-predominant age-related TDP-43 encephalopathy (Neumann et al., 2006; Nelson et al., 2019). There are many lines of evidence suggesting that cytoplasmic aggregation of TDP-43 may play a key role in neurodegeneration (Hergesheimer et al., 2019). Here we report, for the first time, that neuronal inhibition of NF-κB signaling by expression of IκB-SR was able to mitigate the cytoplasmic TDP-43 proteinopathy and behavioral deficits in transgenic models of ALS/FTLD overexpressing TDP-43^A315T^ or TDP-43^G348C^ mutants.

In a previous study, we reported an interaction between TDP-43 and p65-NF-κB in the spinal cord from ALS cases and from TDP-43 transgenic mice (Swarup et al., 2011b). Our results suggested that such abnormal TDP-43/p65 interaction in ALS can enhance NF-κB activity. Interestingly, the pro-oncoprotein FUS, another RNA-binding protein was also shown to bind p65-NF-κB and to serve as its coactivator (Uranishi et al., 2001). These findings led us to propose that NF-κB signaling in neurons may constitute a therapeutic target for ALS disease (Swarup et al., 2011b). So, to address the role of neuronal NF-κB activity, we generated a transgenic mouse line expressing IκB-SR under the control of neurofilament Nefh gene promoter. After crossing the IκB-SR mice with TDP-43^A315T^ and TDP-43^G348C^ mice,the resulting double-transgenic mice exhibited, as expected, a reduced neuronal activation of NF-κB activity compared with single-transgenic TDP-43^A315T^ and TDP-43^G348C^ mice. Thus, the levels of phospo-p65-NF-κB immunostaining were significantly lower in nuclei of spinal neurons from the double-transgenic mice coexpressing IκB-SR compared with single-transgenic littermates TDP-43^A315T^ or TDP-43^G348C^ (Fig. 2). Moreover, inhibition of NF-κB signaling by IκB-SR in double-transgenics resulted in a loss of interaction of TDP-43 with p65 NF-κB, unlike the coimmunoprecipitation of TDP-43 with p65 NF-κB in spinal cord extracts of TDP-43^G348C^ mice (Fig. 2*H*).

A remarkable outcome of neuronal inhibition of NF-κB activity by expression of IκB-SR in TDP-43^A315T^ or TDP-43^G348C^ mice was the substantial reduction of C:N ratio of human TDP-43 as well as decrease in levels of aggregated TDP-43 recovered in the insoluble fraction of spinal cord extract (Fig. 3). What mechanisms could explain such rescue of TDP-43 proteinopathy by inhibition of NF-κB activity? In normal conditions, TDP-43 shuttles between the nucleus and cytoplasm mediating physiological functions at either location (Ayala et al., 2008; Guo and Shorter, 2017). However, structural modifications may cause the protein to accumulate in the cytoplasm with ensuing neurotoxicity (Barmada et al., 2010). Alterations at the RNA recognition motif 1 (RRM1) domain of TDP-43 by oxidation can induce its aggregation and mislocalization in the cytoplasm (Chang et al., 2013; Shodai et al., 2013; Garnier et al., 2017). Moreover, normal RNA binding to this domain is necessary to prevent TDP-43 misfolding, oligomerization, and subsequent accumulation (Zacco et al., 2018). There is a possibility that abnormal binding of p65NF-κB to the RRM1 domain of TDP-43 could interfere with normal protein folding or with RNA binding, resulting in TDP-43 aggregation in the cytoplasm. Accordingly, NF-κB inhibition would restore in part normal TDP-43 interactions. This view is supported by our recent report that single-chain antibodies targeting the RRM1 domain blocked interaction of TDP-43 with p65NF-κB, and they restored normal TDP-43 functions (Pozzi et al., 2019). Another likely mechanism by which NF-κB inhibition may contribute to the clearance of excess TDP-43 levels in the neuronal cytoplasm is by the induction of protein degradation pathways, namely, the ubiquitin-proteasome system and autophagy. Studies have shown that soluble form of cytosolic TDP-43 is primarily targeted for degradation by the ubiquitin-proteasome system, whereas insoluble oligomeric forms tend to be cleared by autophagy (Scotter et al., 2014). NF-κB inhibition can lead to activation of the c-Jun N-terminal kinase pathway that ultimately facilitates the dissociation of Beclin1 from Bcl-2. thereby promoting autophagy (Djavaheri-Mergny et al., 2006; Gao et al., 2016). Consistent with this view, in the double-transgenic TDP-43 mice coexpressing IκB-SR mice, there was an upregulation of autophagy markers Beclin1 and LC3b, which could possibly explain a clearance of cytoplasmic TDP-43 accumulations in spinal neurons.

The mitigation of TDP-43 proteinopathy by neuronal NF-κB inhibition in the double-transgenic mice was associated with a partial rescue of large spinal motor neurons at 1 year of age and with improved motor performance (Fig. 4). Furthermore, the neuronal expression of IκB-SR ameliorated the cognitive deficits of mice expressing TDP-43 mutations as determined by the novel object recognition assay and passive avoidance test (Fig. 5). These results are in line with a previous report that conditional suppression of mutant TDP-43 in a transgenic mouse model resulted in amelioration of cognitive performance (Ke et al., 2015). Cognitive decline during aging is a characteristic feature of transgenic mice expressing mutant TDP-43 mice, which is reminiscent of human FTLD (Swarup et al., 2011a).

We tested the effects of neuronal IκB-SR expression in the SOD1^G93A^ mouse model which develop a severe form of ALS-like disease with accumulation of misfolded SOD1 and massive death of spinal motor neurons (Dion et al., 2009). To date, studies on the role of NF-κB in the SOD1^G93A^ model have been focused primarily to glial responses (Li et al., 2011; Kawabe et al., 2014; Ouali Alami et al., 2018; Schiaffino et al., 2018). Although inhibition of NF-κB activation in astrocytes did not confer neuroprotection in mutant SOD1 mice (Crosio et al., 2011), suppression of NF-κB in microglia extended life span and delayed onset of disease phenotypes (Frakes et al., 2014). Here, our results revealed that selective suppression of neuronal NF-κB activation in SOD1^G93A^ caused a significant extension in their life expectancy (Fig. 8*A*). At all stages of disease, the levels of misfolded SOD1 species were reduced, supporting again the view that neuronal NF-κB suppression may induce neuroprotection by clearance of excess misfolded proteins via induction of autophagy. Thus, at 125 d of age, there were significantly more motor neurons and ChAT reactivity in the spinal cord of double-transgenic IκB-SR;SOD1^G93A^ mice compared with SOD1^G93A^ mice (Fig. 9).

In conclusion, our results suggest a key role for neuronal NF-κB activity in ALS pathogenesis associated with TDP-43 proteinopathy or SOD1 misfolding. It is widely acknowledged that deregulated NF-κB signaling may contribute to acute neurodegenerative conditions, such as cerebral ischemia and traumatic brain injury (Harari and Liao, 2010) as well as chronic neurodegenerative disorders, such as Alzheimer's disease (Jones and Kounatidis, 2017), Parkinson's disease (Flood et al., 2011), Huntington's disease (Bečanović et al., 2015), and ALS (Sako et al., 2012). However, an involvement of NF-κB activity in diseases is commonly referring to pathogenic pathways of immune cells or glial cells. Our finding that suppression of neuronal NF-κB activity with IκB-SR alleviated proteinopathies and behavioral phenotypes in three mouse models of ALS or FTLD suggests that the NF-κB pathway in neurons constitutes a promising therapeutic target. Thus, preclinical studies with ALS models are needed to test pharmacological inhibitors of the neuronal NF-κB signaling pathway for their potency to confer neuroprotection and, especially, to mitigate TDP-43 pathology.
