# Supplementary material for: Mitigation of ALS Pathology by Neuron-Specific Inhibition of Nuclear Factor Kappa B Signaling
Source: J Neurosci. 2020 Jun 24;40(26):5137–54. doi: 10.1523/JNEUROSCI.0536-20.2020 (PMC7314413; doi:10.1523/JNEUROSCI.0536-20.2020)
Supplement: Table 1-1 [file ns-JN-RM-0536-20-s01.docx]

Table 1-1: Comparison between reflex scoring of IκB-SR;SOD1^G93A^ vs SOD1^G93A^ (Figure 8D)

| Age (in days) | *P* value |
| --- | --- |
| 141 | **0.088374** |
| 142 | **0.065396** |
| 143 | **0.033122** |
| 144 | **0.040718** |
| 145 | **0.0243** |
| 146 | **0.031437** |
| 147 | **0.031437** |
| 148 | **0.048639** |
| 149 | **0.040284** |
| 150 | **0.005576** |
| 151 | **0.007446** |
| 152 | **0.006464** |
| 153 | **0.009698** |
| 154 | **0.014878** |
| 155 | **0.005328** |
| 156 | **0.010296** |
| 157 | **0.017912** |
| 158 | **0.022184** |
| 159 | **0.027559** |
| 160 | **0.031168** |
| 161 | **0.014623** |
