# Supplementary figures and images for: Mitigation of ALS Pathology by Neuron-Specific Inhibition of Nuclear Factor Kappa B Signaling
Source: J Neurosci. 2020 Jun 24;40(26):5137–54. doi: 10.1523/JNEUROSCI.0536-20.2020 (PMC7314413; doi:10.1523/JNEUROSCI.0536-20.2020)

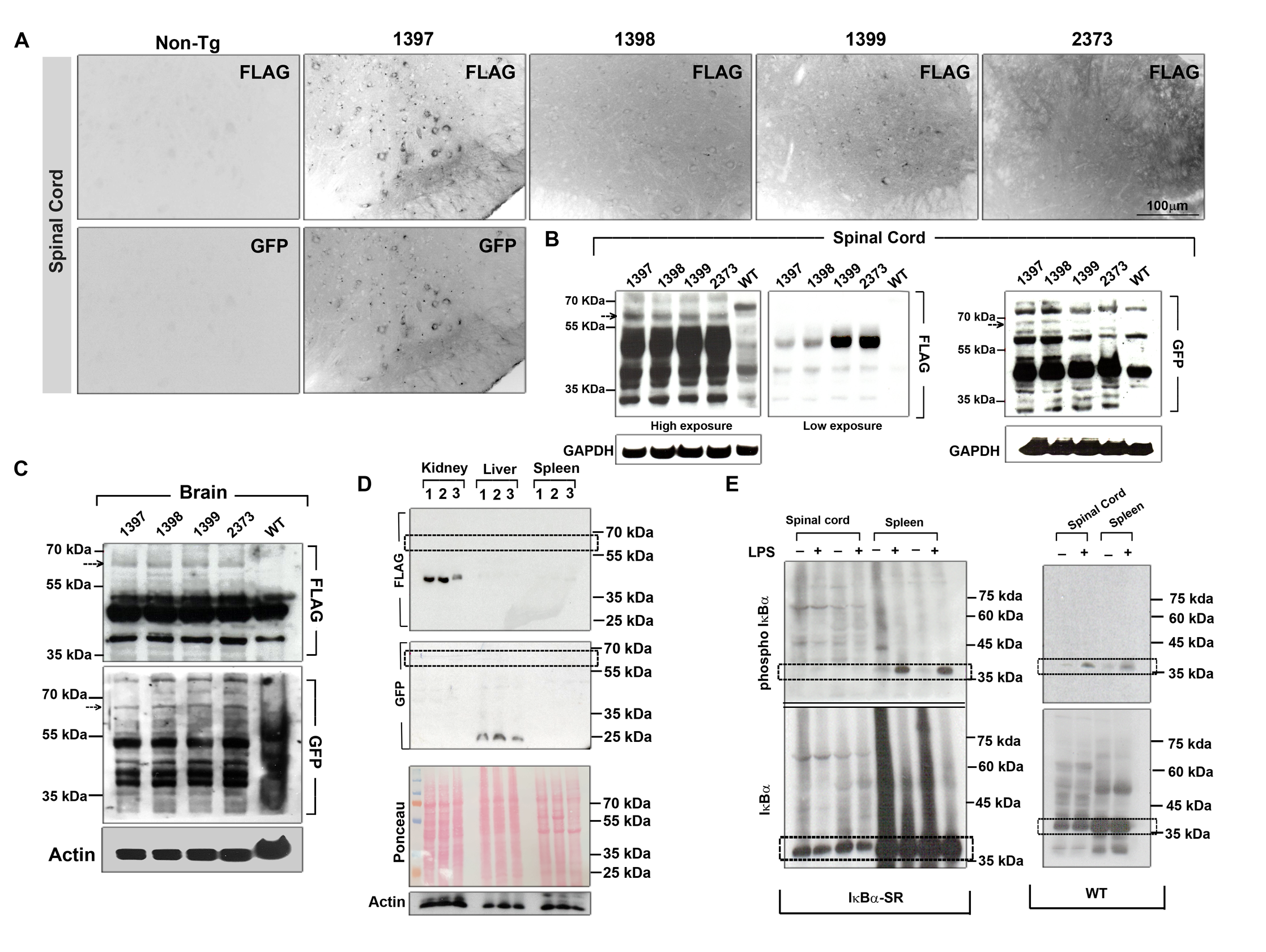

Supplement: Figure 1-1 [file ns-JN-RM-0536-20-s02.tif]

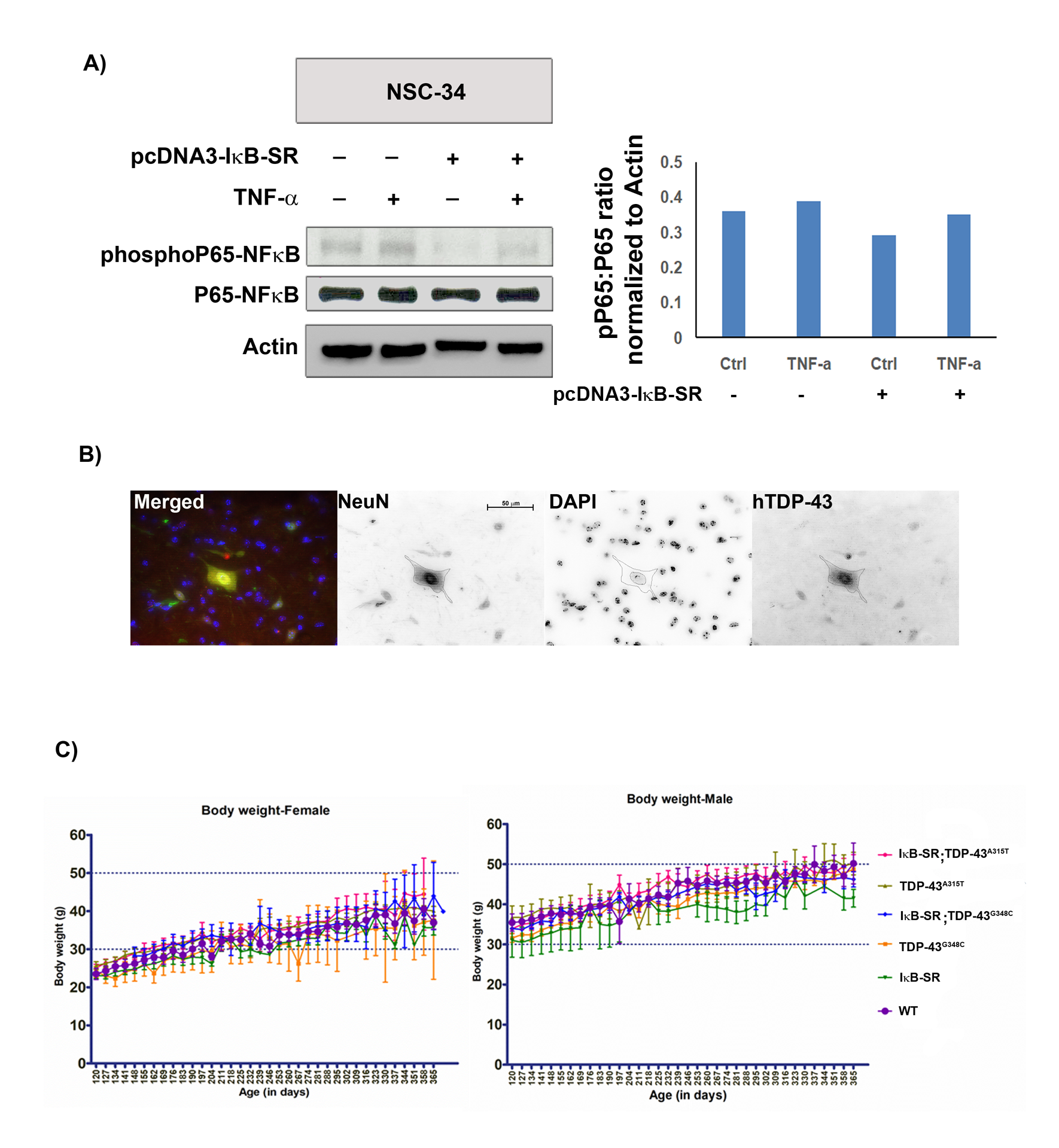

Supplement: Figure 2-2 [file ns-JN-RM-0536-20-s03.tif]
